# Suppression of RNA Silencing by a Plant DNA Virus Satellite Requires a Host Calmodulin-Like Protein to Repress *RDR6* Expression

**DOI:** 10.1371/journal.ppat.1003921

**Published:** 2014-02-06

**Authors:** Fangfang Li, Changjun Huang, Zhenghe Li, Xueping Zhou

**Affiliations:** 1 State Key Laboratory of Rice Biology, Institute of Biotechnology, Zhejiang University, Hangzhou, China; 2 State Key Laboratory for Biology of Plant Diseases and Insect Pests, Institute of Plant Protection, Chinese Academy of Agricultural Sciences, Beijing, China; University of California Riverside, United States of America

## Abstract

In plants, RNA silencing plays a key role in antiviral defense. To counteract host defense, plant viruses encode viral suppressors of RNA silencing (VSRs) that target different effector molecules in the RNA silencing pathway. Evidence has shown that plants also encode endogenous suppressors of RNA silencing (ESRs) that function in proper regulation of RNA silencing. The possibility that these cellular proteins can be subverted by viruses to thwart host defense is intriguing but has not been fully explored. Here we report that the *Nicotiana benthamiana* calmodulin-like protein Nbrgs-CaM is required for the functions of the VSR βC1, the sole protein encoded by the DNA satellite associated with the geminivirus *Tomato yellow leaf curl China virus* (TYLCCNV). *Nbrgs-CaM* expression is up-regulated by the βC1. Transgenic plants over-expressing *Nbrgs-CaM* displayed developmental abnormities reminiscent of βC1-associated morphological alterations. Nbrgs-CaM suppressed RNA silencing in an *Agrobacterium* infiltration assay and, when over-expressed, blocked TYLCCNV-induced gene silencing. Genetic evidence showed that *Nbrgs-CaM* mediated the βC1 functions in silencing suppression and symptom modulation, and was required for efficient virus infection. Moreover, the tobacco and tomato orthologs of *Nbrgs-CaM* also possessed ESR activity, and were induced by betasatellite to promote virus infection in these *Solanaceae* hosts. We further demonstrated that βC1-induced Nbrgs-CaM suppressed the production of secondary siRNAs, likely through repressing *RNA-DEPENDENT RNA POLYMERASE 6* (*RDR6*) expression. *RDR6*-deficient *N. benthamiana* plants were defective in antiviral response and were hypersensitive to TYLCCNV infection. More significantly, TYLCCNV could overcome host range restrictions to infect *Arabidopsis thaliana* when the plants carried a *RDR6* mutation. These findings demonstrate a distinct mechanism of VSR for suppressing PTGS through usurpation of a host ESR, and highlight an essential role for RDR6 in RNA silencing defense response against geminivirus infection.

## Author Summary

In plants, RNA silencing plays a key role in developmental regulation and antiviral defense. To successfully infect their hosts, plant viruses encode silencing suppressors (VSRs) as counter-defense measures. These VSRs function to disable host antiviral RNA silencing defenses through various mechanisms that are not well understood. Here we report that a host calmodulin-like protein called Nbrgs-CaM, which appears to be an endogenous suppressor of RNA silencing, plays essential roles in suppression of RNA silencing and induction of symptoms by the VSR βC1, the sole protein encoded by a geminivirus-associated DNA satellite. The *Nbrgs-CaM* was up-regulated by Tomato yellow leaf curl China geminivirus (TYLCCNV)-encoded VSR βC1 upon virus infection or stable expression via a transgene. Further analyses revealed that up-regulation of *Nbrgs-CaM by* βC1 suppressed RNA silencing likely through repressing the expression of *RNA-DEPENDENT RNA POLYMERASE 6* (*RDR6*). We have demonstrated that RDR6-mediated RNA silencing plays an important role in antiviral defense in *Nicotiana benthamiana* and confers host range restriction against TYLCCNV infection on *Arabidopsis thaliana*. Our study suggests that exploiting a cellular suppressor can be an efficient mechanism for viruses to counteract host RNA silencing defense response.

## Introduction

Viruses are obligate molecular parasites that have limited coding capacity and depend on host resources to survive. To successfully infect their hosts, viruses have evolved strategies to exploit cellular functions for multiplication, as well as mechanisms to evade or subvert elaborate host defense mechanisms. In plants, RNA silencing, also called post-transcriptional gene silencing (PTGS), represents an important antiviral immunity mechanism that operates in a sequence-specific manner. Double-stranded RNAs (dsRNAs) derived from virus replication products serve as pathogen-associated molecular patterns for defense recognition and are processed into primary small interfering RNAs (siRNAs) of 21 to 25 nucleotides (nt) by ribonuclease III-like enzymes, termed DICER-like proteins (DCLs) in plants. The siRNAs then interact with members of the ARGONAUTE (AGO) family of proteins to form RNA-induced silencing complexes (RISCs), which use the siRNAs as guides to target cognate viral RNAs for cleavage. In plants, fungi and nematodes, viral RNAs or their cleavage products can serve as templates for host RNA-dependent RNA-polymerases (RDRs) to produce dsRNAs that subsequently generate secondary siRNA by DCLs cleavage [1], [2]. Two RDRs have been implicated in plant virus defense [2]–[4]: Tobacco RDR1 is induced by salicylic acid or virus infection and functions to restrict systemic spread of *Tobacco mosaic virus* (TMV) and *Potato virus X* (PVX) [5]; in *Arabidopsis thaliana*, efficient RNA silencing also requires RDR6 and its dsRNA-binding partner, Suppressor of Gene Silencing 3 (SGS3), to amplify viral siRNAs that allow plants to mount effective defense response against virus infection [6]–[10]. Likewise, *Nicotiana benthamiana* plants with reduced RDR6 levels develop hypersusceptibility to some RNA viruses [8], [11]–[14], emphasizing the important role of RDR6 in antiviral defense.

Despite the potency of RNA silencing in antiviral defenses, plant viruses still systemically infect diverse plant species and cause diseases. Most, if not all, plant viruses have evolved mechanisms to counterattack RNA silencing by encoding proteins termed viral suppressors of RNA silencing (VSRs) [1], [15], [16]. Various VSRs often share little sequence similarity and target different steps in the RNA silencing pathway. A common strategy employed by some VSRs is to bind to dsRNA or siRNA duplexes, thereby preventing the sensing and dicing of dsRNA trigger, or interfering with the incorporation of siRNA into RISC [17]–[20]. Other suppressors directly target component of dicing machinery. One such example is P6 of *Cauliflower mosaic virus* (CaMV), which interferes with viral siRNAs processing by interacting with dsRNA-binding protein 4, an essential partner of the antiviral DCL4 [21]. Alternatively, some VSRs, such as 2b of *Cucumber mosaic virus* (CMV), p38 of *Turnip crinkle virus* (TCV) and P0 of poleroviruses, either inhibit AGO functions [22], [23], or target AGO proteins for degradation [24], [25].

Several studies have shown that some VSRs suppress PTGS indirectly by affecting cellular regulators of the small RNA pathway. For instance, p19 of *Cymbidium ring spot virus* represses the AGO1-directed antiviral response by specific induction of miR168, which in turn, negatively regulates AGO1 mRNA levels [26]. Suppression of PTGS by HC-Pro of *Tobacco etch virus* (TEV) is mediated by the tobacco calmodulin-like protein rgs-CaM, the first identified endogenous suppressor of RNA silencing (ESR) [27]. In addition, an ethylene-induced transcription factor RAV2 in *Arabidopsis* is needed for suppression of primary RNA silencing by two unrelated VSRs, namely HC-Pro of *Turnip mosaic virus* (TuMV) and p38 of TCV. Although RAV2 itself has not been shown to directly suppress RNA silencing, it is necessary for TuMV HC-Pro to induce the expression of several putative ESRs including *AtCML38* (Calmodulin-like protein 38), a closely related homolog of rgs-CaM in *Arabidopsis* [28]. Calmodulins or calmodulin-like proteins are small acidic proteins that contain varied numbers of the ‘EF-hand’ motif. These proteins sense and decode cellular calcium (Ca^2+^) cation signals through high-affinity binding of their EF-hand domains to Ca^2+^, which induces a conformation change in the proteins and exposes their hydrophobic surfaces. The activated calmodulins then interact with many downstream target proteins to modulate their activities in diverse cellular functions. Alternatively, some of the calmodulin/Ca^2+^ complexes may function directly as transcription regulators to control the expression of downstream effectors [29], [30]. Plants have evolved a diversity of calmodulins and calmodulin-like proteins that play a vital role in response to development cues and environmental stimuli [29]. The identification of the tobacco rgs-CaM as an ESR indicates that calmodulin-like proteins are involved in fine-tuning the functions of the RNA silencing pathway, which has also been implicated in developmental regulation and stress responses [31].

The *Geminiviridae* is a large family of plant viruses that cause economically important crop diseases worldwide. Geminiviruses contain circular, single-stranded DNA (ssDNA) genomes encapsidated in twinned icosahedral particles. [32]. Although ssDNA viruses have no dsRNA phase in their replication cycle, transcripts produced by geminiviruses can both induce and be targeted by cytoplasmic PTGS (reviewed in [33], [34]). Geminivirus-derived small RNAs of 21, 22, and 24 nt have been detected in infected hosts and all four DCLs in *Arabidopsis* have been implicated in the production of geminiviral siRNAs [35]. Studies of virus induced gene silencing (VIGS) triggered by *Cabbage leaf curl virus* (CaLCuV), a member of the *Begomovirus* genus belonging to the *Geminiviridae* family, have revealed requirements for RDR6 and SGS3 in CaLCuV-induced RNA silencing of an endogenous gene [36]. RDR6 and SGS3 are thought to convert geminivirus-derived transcripts into dsRNAs that induce RNA silencing [36]. Consequently, several proteins encoded by geminiviruses are able to suppress PTGS (reviewed in [33]), although the mechanisms in many cases are not fully understood.

Betasatellites represent novel DNA molecules that are associated with some monopartite begomoviruses in the *Geminiviridae* family including *Tomato yellow leaf curl China virus* (TYLCCNV) [37], [38]. While able to infect their hosts alone, these monopartite begomoviruses are often incapable of inducing typical symptoms, and require betasatellites for accumulation to high titers and elicitation of disease symptoms [39], [40]. The betasatellite contains a single open reading frame (βC1) that encodes a symptom determinant [41]–[44]. Earlier studies have shown that βC1 proteins are potent PTGS suppressors [43]–[49], but their modes of action have not been elucidated. In the present study, we have demonstrated that the *N. benthamiana* calmodulin-like protein, designated “regulator of RNA silencing” (Nbrgs-CaM) is up-regulated by βC1 of *Tomato yellow leaf curl China betasatellite* (TYLCCNB) and that Nbrgs-CaM is required for both PTGS suppression and symptom induction by TYLCCNB βC1. We also have determined that Nbrgs-CaM suppressed PTGS likely acting through repressing the expression of *NbRDR6*, an important component in the anti-geminiviral RNA silencing pathway. These data, together with previous findings with TEV HC-Pro [27], suggest that cellular regulators of RNA silencing can be subverted by evolutionarily divergent plant viruses to counteract antiviral defenses.

## Results

### TYLCCNV betasatellite-encoded βC1 up-regulates the expression of a host calmodulin-like protein

**Figure 1:**
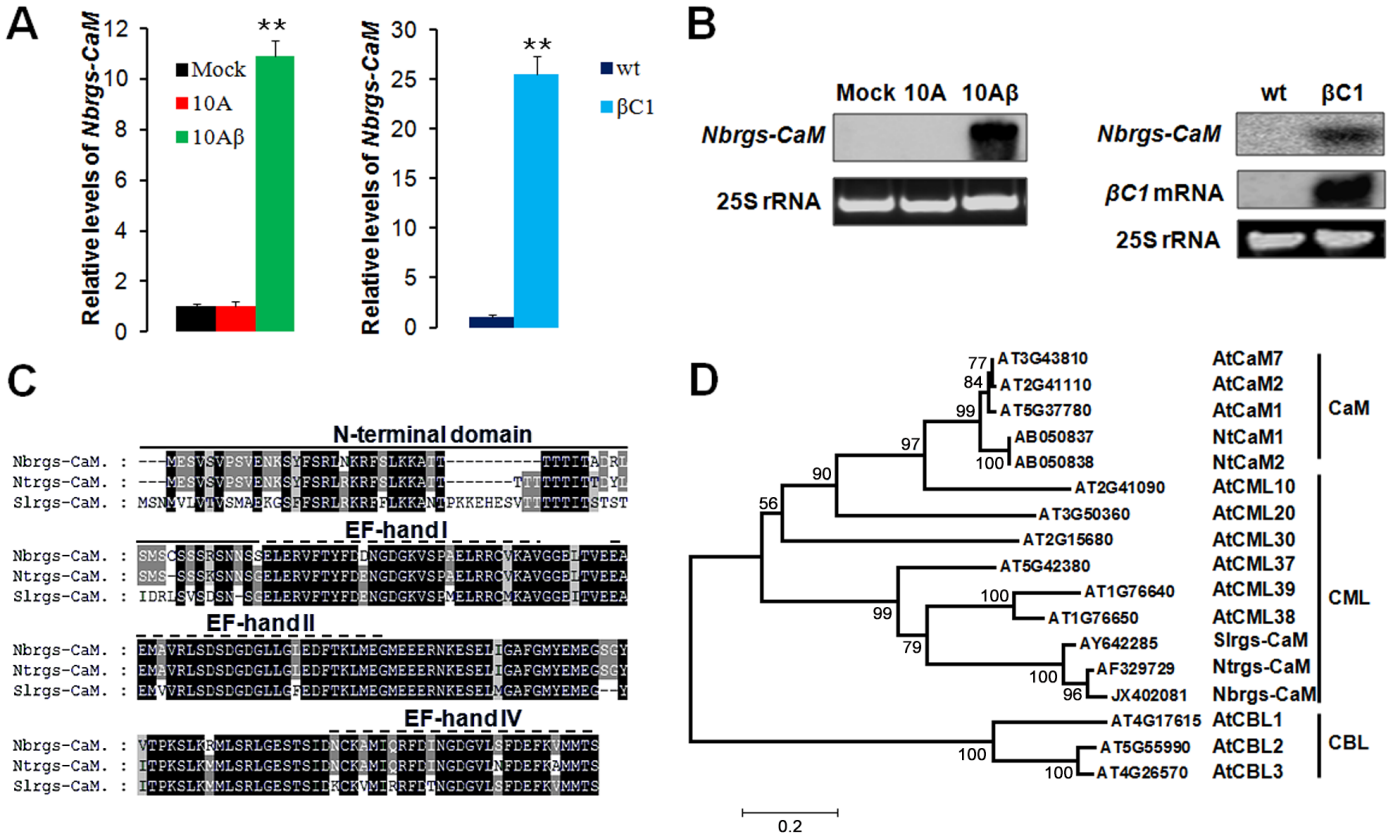
Induction of *Nicotiana benthamiana* calmodulin-like protein Nbrgs-CaM by TYLCCNB βC1. (**A**) RT-qPCR analyses of *Nbrgs-CaM* mRNA levels in *N. benthamiana* plants infected by TYLCCNV (10A) alone or together with betasatellite (10Aβ), or with a*grobacterium* carrying an empty vector (Mock) (left bar graph) at 21 dpi, and in βC1 transgenic plants (βC1) and nontransgenic control plants (wt) at 6 to 7-leaf period (right bar graph). Each mean value was derived from three independent experiments (*n* = 9) and normalized to mRNA of *NbGAPDH* that served as an internal standard. The relative mRNA levels of *Nbrgs-CaM* in mock plants (left bar graph) or wt plants (right bar graph) is arbitrarily set as 1. Values are means ± SD. The data were analyzed using one-way ANOVA followed by Student's *t* test. Asterisk indicates significant differences between genotypes or treatments (**P≤0.01). (**B**) Northern blot analyses of the *Nbrgs-CaM* and *βC1* mRNA levels in plants described in (**A**). Ethidium bromide staining of 25S ribosomal RNA (rRNA) was used to confirm equal RNA loading. (**C**) Pairwise alignment of rgs-CaMs from *N. benthamiana* (Nbrgs-CaM), *N. tabacum* (Ntrgs-CaM) and *Solanum lycopersicum* (Slrgs-CaM) using ClustalX2 software. The N-terminal extension specific for rgs-CaM is denoted by a solid line above the sequences, whereas the three EF-hand motifs characteristic of calmodulins are illustrated by the dotted lines. (**D**) Phylogenetic tree showing the relationship between the three rgs-CaMs and selective calcium-binding proteins from *Arabidopsis* (At) and tobacco (Nt). Numbers represent systemic names or GenBank accession numbers for the corresponding genes. Sequence alignments were performed in ClustalX2 and tree construction was conducted in MEGA5.1 using the Neighbour Joining method. The bootstrap values are shown near the internal nodes. CaM: calmodulin; CML: calmodulin-like protein; CBL: calcineurin B-like protein. The scale bar represents the number of changes per site.

To gain more insights into the mode of action of TYLCCNB βC1 in PTGS suppression, whole genome tiling microarrays were used to probe the changes of transcriptome profile in response to TYLCCNB. The global gene expression patterns of *N. benthamiana* infected with TYLCCNV isolate Y10 (hereafter referred to 10A) were compared to infections with TYLCCNV and the associated TYLCCNB betasatellite (hereafter referred to 10Aβ). The most pronounced of the differentially expressed genes, was an *N. benthamiana* ortholog of tobacco calmodulin-like protein, which has been previously identified as an endogenous regulator of gene silencing (rgs-CaM) that is induced by the potyvirus-encoded VSR HC-Pro [27]. Relative transcript quantification by reverse transcription quantitative real-time PCR (RT-qPCR) verified that *N. benthamiana rgs-CaM* (*Nbrgs-CaM*) is substantially up-regulated in 10Aβ-infected plants as compared to plants infected by 10A alone or mock-treated plants (Fig. 1A). Northern blots analyses indicated that the levels of *Nbrgs-CaM* mRNA are low in mature leaves of mock-treated plants and of 10A-infected plants, but are enhanced greatly by 10Aβ infections (Fig. 1B). To determine whether βC1, the only protein known to be encoded by TYLCCNB, is responsible for the enhanced expression of *Nbrgs-CaM*, we measured the mRNA levels of *Nbrgs-CaM* in transgenic *N. benthamiana* plants expressing *βC1* under the control of the constitutive CaMV 35S promoter [39]. Levels of *Nbrgs-CaM* mRNA in the *βC1* transgenic plants were over 20-fold higher than those of wild-type (wt) plants (Fig. 1A–B). In addition, in other *Solanaceae* hosts, TYLCCNV infections containing the betasatellite also exhibited up-regulated expression of the *rgs-CaM* orthologs in tobacco (*Ntrgs-CaM*) and tomato (*Slrgs-CaM*) (Fig. S1).

EST sequences of *Nbrgs-CaM* were aligned to *Ntrgs-CaM* and *Slrgs-CaM* sequences obtained from the Genbank database, and primers were designed to amplify the full-length genes encoding the proteins. Sequencing revealed that the *Nbrgs-CaM* open reading frame (ORF) is 573-nt (GenBank accession number JX402081), and Blast searches against the *N. benthamiana* transcriptome database (http://benth-web-pro-1.ucc.usyd.edu.au/blast/blast.php) demonstrated that the ORF had a 100% identity to the Nbv3K725607008 unigene transcript. Sequence analysis also revealed that *Nbrgs-CaM* encodes a 188-amino acid (aa) calcium-binding protein, which has the highest similarity (94% and 80% aa identities) to Ntrgs-CaM and Slrgs-CaM ORF, respectively. These rgs-CaM orthologs contain a ∼50-aa N-terminal domain, in addition to a calmodulin (CaM) domain in the C-terminal region that has three characteristic EF-hand calcium binding motifs (EF I, II and IV) (Fig. 1C). A phylogenetic tree was constructed to compare the evolutionary relationships between the rgs-CaMs and representative calmodulins (CaM), calmodulin-like (CML) proteins and calcineurin B-like (CBL) proteins from *Arabidopsis* and *Nicotiana* spp. The Rgs-CaMs are clustered with *Arabidopsis* CMLs, with the closest relationships to CML38 and CML39. The three Rgs-CaMs are only distantly related to two CaMs from tobacco (NtCaM1 and NtCaM2), which formed a separate branch with CaMs from *Arabidopsis* (Fig. 1D).

### *Nbrgs-CaM*-overexpression in *N. benthamiana* plants phenocopies βC1-transgenic plants

**Figure 2:**
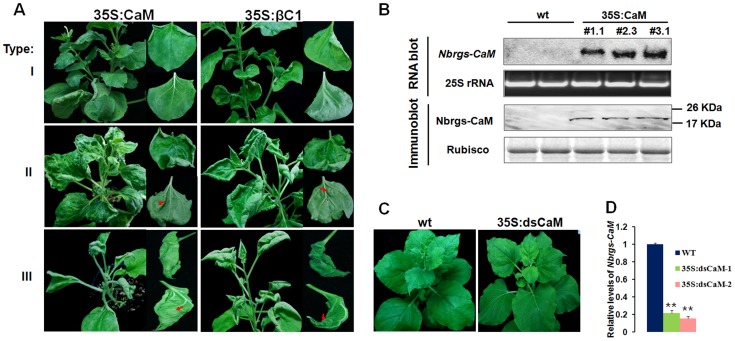
Ecotopic expression of *Nbrgs-CaM* in *Nicotiana benthamiana* phenocopys βC1 transgenic plants. (**A**) Parallel comparisons of phenotypes in Nbrgs-CaM and βC1 transgenic *N. benthamiana* plants. Representative leaves of type I, type II and type III 35S:CaM transgenic plants with varied phenotypic severities were shown on the left panel with the close-up views of the adaxial and abaxial side of upward-curled leaves. βC1 transgenic plants were shown in parallel on the right panels. The arrows indicate outgrowth tissues on the abaxial leaf surfaces of Nbrgs-CaM and βC1 transgenic plants. (**B**) Analysis of *Nbrgs-CaM* mRNA and protein levels in nontransgenic (wt) and three *Nbrgs-CaM* transgenic lines (35S:CaM). Equal loading of proteins and total RNA were confirmed by Coomassie blue staining of Rubisco large subunit and ethidium bromide staining of 25S rRNA, respectively. (**C** and **D**) Phenotype of 35S:dsCaM transgenic *N. benthamiana* (**C**) and levels of *Nbrgs-CaM* analyzed by RT-qPCR in 35S:dsCaM-1, 35S:dsCaM-2 transgenic lines at 6–7 leaf period (**D**). Values are means ± SD. Nine individual plants per genotype were used in each of the measurements. The asterisk indicates significant differences between genotypes analyzed by Student *t* test (**P≤0.01).

To investigate functional links between Nbrgs-CaM and βC1, we generated transgenic *N. benthamiana* lines over-expressing *Nbrgs-CaM* under the control of the CaMV 35S promoter (35S:CaM). Many of the Nbrgs-CaM primary transgenic plants displayed developmental abnormities that could be grouped into three types based on phenotypic severity. Type I plants developed mild morphological alterations with slightly upward curled leaves. Type II plants showed moderate upward leaf curling and small interveinal outgrowths from abaxial leaf surfaces (Fig. 2A). The upper leaves of the type I and II plants usually appeared normal (Fig. 2A), and these plants produced fertile inflorescence. Type III plants had severely distorted, upward curling leaves and more extensive tissues outgrowth (Fig. 2A). The upper leaves of Type III plants maintained the severe curling syndrome and most of these plants failed to produce inflorescences or produced only sterile flowers. Overall the phenotypes of 35S:CaM plants were strikingly similar to those of *N. benthamiana* transgenic plants expressing *βC1* (35S:βC1) [39]. Both the 35S:CaM and 35S:βC1 transgenic plants developed similar abnormities including upward leaf curling, vein swelling and outgrowth of tissues (compare left panels with right panels in Fig. 2A), albeit several 35S:CaM plants lines showed a chlorotic mottling on leaves that was not routinely observed in 35S:βC1 plants (Fig. 2A, middle panel). Three independent transgenic lines #1.1, #2.3 and #3.1, which represented type I, II and III phenotype plants, respectively, were analyzed for transgene expression by Northern and Western blotting. High levels of Nbrgs-CaM accumulated in these transgenic lines compared to undetectable levels in nontransgenic (wt) plants (Fig. 2B). Although plants with severer phenotypes appeared to have slightly higher levels of *Nbrgs-CaM* mRNA and protein (Fig. 2B), a direct correlation between transgene expression and symptom severity was not evident. Nevertheless, the phenotypic resemblance between 35S:CaM and 35S:βC1 plants suggests that both genes may have functional similarities. For subsequent studies, line #1.1 was used unless otherwise stated due to its high levels of transgene expression and lack of severe phenotype in the plants.

Transgenic plants with reduced expression of endogenous *Nbrgs-CaM* were also generated by transforming *N. benthamiana* plants with a hairpin RNA interference (RNAi) construct under the control of CaMV 35S promoter (35S:dsCaM). These 35S:dsCaM plants appeared to be normal without discernible developmental defects (Fig. 2C). Moreover, *Nbrgs-CaM* transcripts in wt and 35S:dsCaM plants were low in abundance and difficult to detect by Northern blotting (data not shown), although RT-qPCR showed that RNAi had reduced the *Nbrgs-CaM* mRNAs to approximately 20% of wt levels (Fig. 2D).

### Nbrgs-CaM suppresses PTGS and blocks geminivirus-induced gene silencing

**Figure 3:**
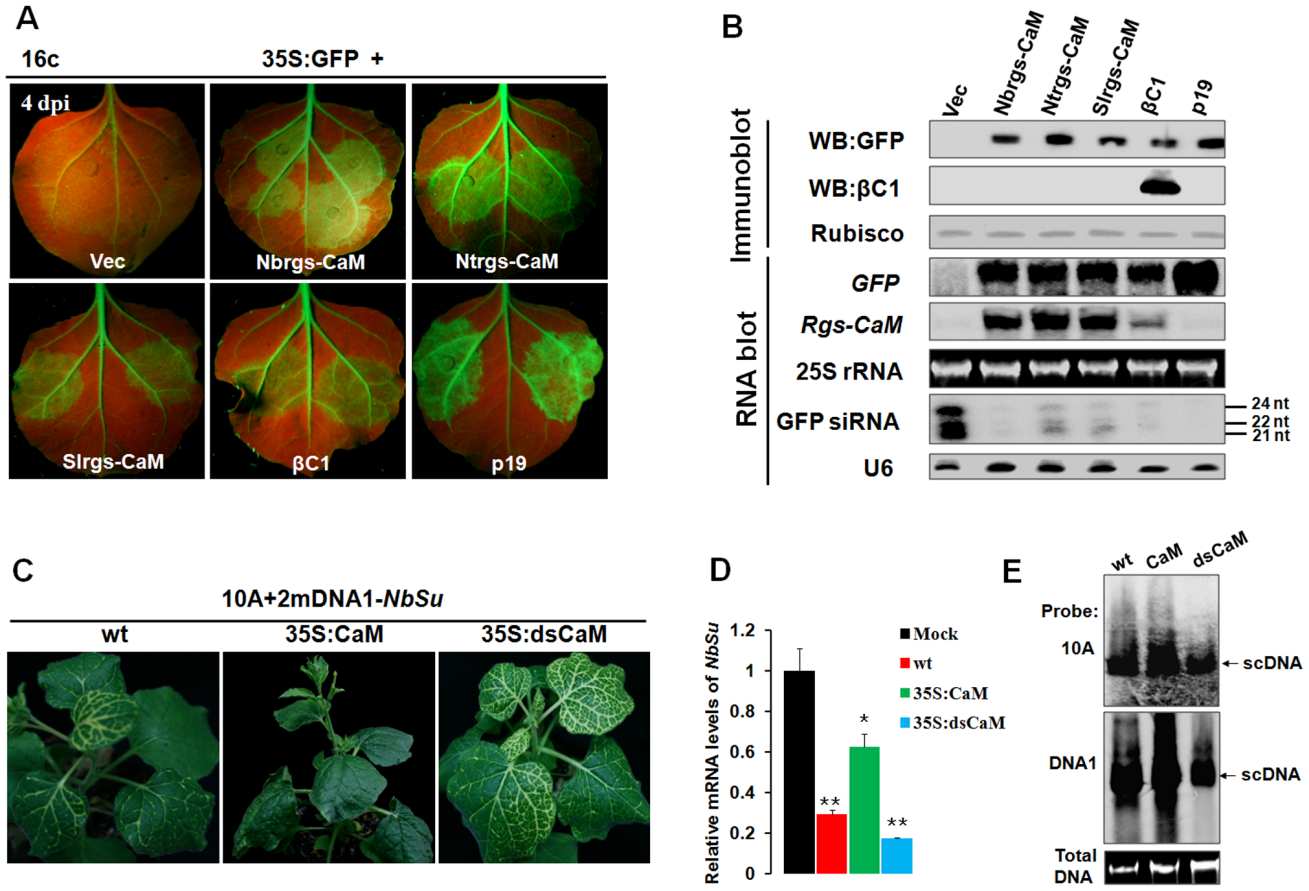
Nbrgs-CaM suppresses PTGS of GFP and VIGS of an endogenous gene. (**A**) Suppression of GFP silencing in *Nicotiana benthamiana* line 16c. Leaf patches were co-infiltrated with *Agrobacterium tumefaciens* cultures expressing *GFP* (35S:GFP) and vector control (Vec), *Nbrgs-CaM*, *Ntrgs-CaM*, *Slrgs-CaM*, TYLCCNB *βC1* or TBSV *p19*, and GFP fluorescence were photographed under UV light at 4 dpi. (**B**) Protein and RNA gel blot analyses of GFP silencing in agroinfiltrated leaf samples. GFP or βC1 specific monoclonal antibody was used in immunoblottings. [α-^32^P]-labeled *GFP*- or *Nbrgs-CaM*-specific probe was used in the large RNA blot. Note that the *Nbrgs-CaM*-specific probes also cross-reacts to *Ntrgs-CaM* and *Slrgs-CaM* mRNAs in Northern blots owing to their high sequence similarities. [γ-^32^P] ATP-labeled GFP or U6 oligonucleotides were used as probes in the small RNA blots. The sizes of the 21-, 22- and 24-nt RNAs are indicated to the right of the small RNA panel. Ethidium bromide staining of 25S rRNA and Coomassie blue staining of the large subunit of Rubisco served as loading controls for RNA and protein gels, respectively. (**C**) Phenotypes of *Su* VIGS. Wt, 35S:CaM or 35S:dsCaM *N. benthamiana* plants were infected with the TYLCCNV-derived VIGS vector carrying a portion of the endogenous *Su* gene (10A+2mDNA1-*NbSu*) and photographed at 30 dpi. (**D**) RT-qPCR analysis of *Su* silencing. The relative levels of *Su* mRNA in plants shown in (**C**) were normalized to mRNA of *NbGAPDH* that served as an internal standard. Mock represents relative *Su* mRNA in wt plants infected by the VIGS vector without *Su* insertion (10A+2mDNA1). The relative level of *Su* mRNA in mock plants is arbitrarily set as 1. Error bars represent SD of nine individual plants. Asterisks indicate P values compared with mock-treated wild type plants: *P≤0.05, **P≤0.01 (Student's *t* test). (**E**) Southern blot analysis of viral DNAs in the VIGS vector-infected plants shown in (**C**). Total DNAs were blotted with α-^32^P-labeled probes specific for TYLCCNV (10A) and alphasatellite (DNA1). The migrations of viral supercoiled DNA (scDNA) are indicated to the right of the panel. Total DNA agarose gel was stained with ethidium bromide to show the equal loading.

An earlier study has shown that Ntrgs-CaM suppresses PTGS, representing the first identified ESR in plants [27]. To investigate whether Nbrgs-CaM and its orthologs can suppress PTGS, we employed a transient silencing suppression assay based on the GFP transgenic *N. benthamiana* line 16c [50], [51] in the study. In this assay, *Agrobacterium tumefaciens* cultures harboring a binary vector designed to transiently express sense *GFP* mRNA (35S:GFP) and *Agrobacterium* derivative harboring a candidate suppressor gene were co-infiltrated into leaves of 16c plants. Agroinfiltration of 16c leaves with 35S:GFP and an empty vector (negative control) triggered GFP RNA silencing and resulted in weak GFP fluorescence under long-wavelength UV light at 4-days post infiltration (dpi) (Fig. 3A). As expected, co-infiltration of 35S:GFP with βC1 of TYLCCNB or p19 of *Tomato bushy stunt virus* (TBSV) elicited strong green fluorescence as a consequence of suppression of GFP RNA silencing (Fig. 3A). Expression of *Nbrgs-CaM*, *Ntrgs-CaM* and *Slrgs-CaM* also suppressed GFP silencing as indicated by appearance of green fluorescence in co-infiltrated regions (Fig. 3A). The GFP fluorescence imaging shown in Fig. 3A was also correlated with the accumulation of GFP protein and mRNA as assessed by immunoblot and RNA blot analyses (Fig. 3B). High levels of expression of *Nbrgs-CaM*, *Ntrgs-CaM* and *Slrgs-CaM* in agroinfiltrated leaves were also verified by RNA blotting with *rgs-CaM*-specific probes (Fig. 3B). Notably, transient expression of *βC1* also led to increased accumulation of *Nbrgs-CaM* mRNA compared to undetectable basal levels in vector-infiltrated leaves. By contrast, an unrelated VSR p19 failed to induce *Nbrgs-CaM* expression (Fig. 3B), suggesting that induction of *Nbrgs-CaM* is unlikely a common feature associated with VSRs. Small RNA gel blot analysis revealed that high levels of 21, 22 and 24 nt GFP-specific siRNAs accumulated in the vector control-treated leaves as a result of GFP RNA silencing. In contrast, expressions of the three *rgs-CaMs* and *βC1* markedly decreased the amount of GFP siRNAs (Fig. 3B). Similarly, p19 also inhibited the accumulation of the siRNAs (Fig. 3B) as previously reported [26]. Rgs-CaMs infiltrated leaves had comparable suppression activity to those of βC1 infiltrations, but were weaker than that elicited by p19 (Fig. 3A–B). Overall, these data demonstrate that, as with βC1, all three rgs-CaMs exhibit PTGS suppression activity and inhibit siRNA biogenesis.

To further examine the function of Nbrgs-CaM in RNA silencing suppression in the context of a virus infection, we tested whether Nbrgs-CaM could suppress VIGS by using a previously established VIGS vector based on 10A helper virus in association with a geminivirus alphasatellite derivative (2mDNA1) [52], [53]. The alphasatellite vector (2mDNA1-*NbSu*) was designed to express a fragment of the *N. benthamiana sulfur* (*Su*) reporter gene that encodes a component required for chlorophyll II biosynthesis. The recombinant viral vector (10A+2mDNA1-*NbSu*) triggered *Su* silencing in leaves of infected plants as evidenced by yellow-white leaf spots [52], [53]. In wt *N. benthamiana* infected by the VIGS vector, the typical *Su* silencing phenotype first appeared along the main veins of the newly expanded leaves at 10 dpi, and later expand to the lateral veins to produce a white lattice-like phenotype at 30 dpi (Fig. 3C). However, infected 35S:CaM transgenic line #1.1 failed to develop an *Su* silencing by 30 dpi (Fig. 2C). Even at late stages of infection (40 dpi), only sporadic white spots were observed in some of the newly emerged leaves in 35S:CaM plants (data not shown). On the other hand, *Su* silencing appeared 4 to 5 days earlier in infected *Nbrgs-CaM* RNAi line plants (35S:dsCaM) than in wt plants, and more extensive *Su* silencing was noted at 30 dpi (Fig. 3C). When additional *Nbrgs-CaM* transgenic lines were analyzed, similar lack of or delayed Su silencing phenotypes were observed in 35S:CaM lines, in contrast to more extensive silencing in 35S:dsCaM lines (Fig. S2). Analysis of *Su* mRNA levels in these plants by RT-qPCR also supported the observed silencing phenotypes (Fig. 3D). When compared to endogenous *Su* mRNA level in mock-silenced plants (infection with an empty VIGS vector 10A+2mDNA1), VIGS reduced the *Su* mRNA levels by ∼4-fold in wt plants, and ∼5-fold in 35S:dsCaM plants but less than 2-flod in 35S:CaM plants (Fig. 3D, Fig. S2). Notably, both the 10A helper virus and alphasatellite accumulated to higher levels in 35S:CaM plants and to lower levels in 35S:dsCaM plants, relative to wt plants (Fig. 3E). Therefore, the altered VIGS efficiencies in *Nbrgs-CaM* transgenic lines are not a consequence of differential virus accumulations. These data indicate that the cellular levels of Nbrgs-CaM are negatively correlated with the effectiveness of VIGS in *N. benthamiana*, and support a model that Nbrgs-CaM functions as an endogenous negative regulator of RNA silencing.

### Rgs-CaMs and βC1 target a step upstream of dsRNA generation in PTGS

**Figure 4:**
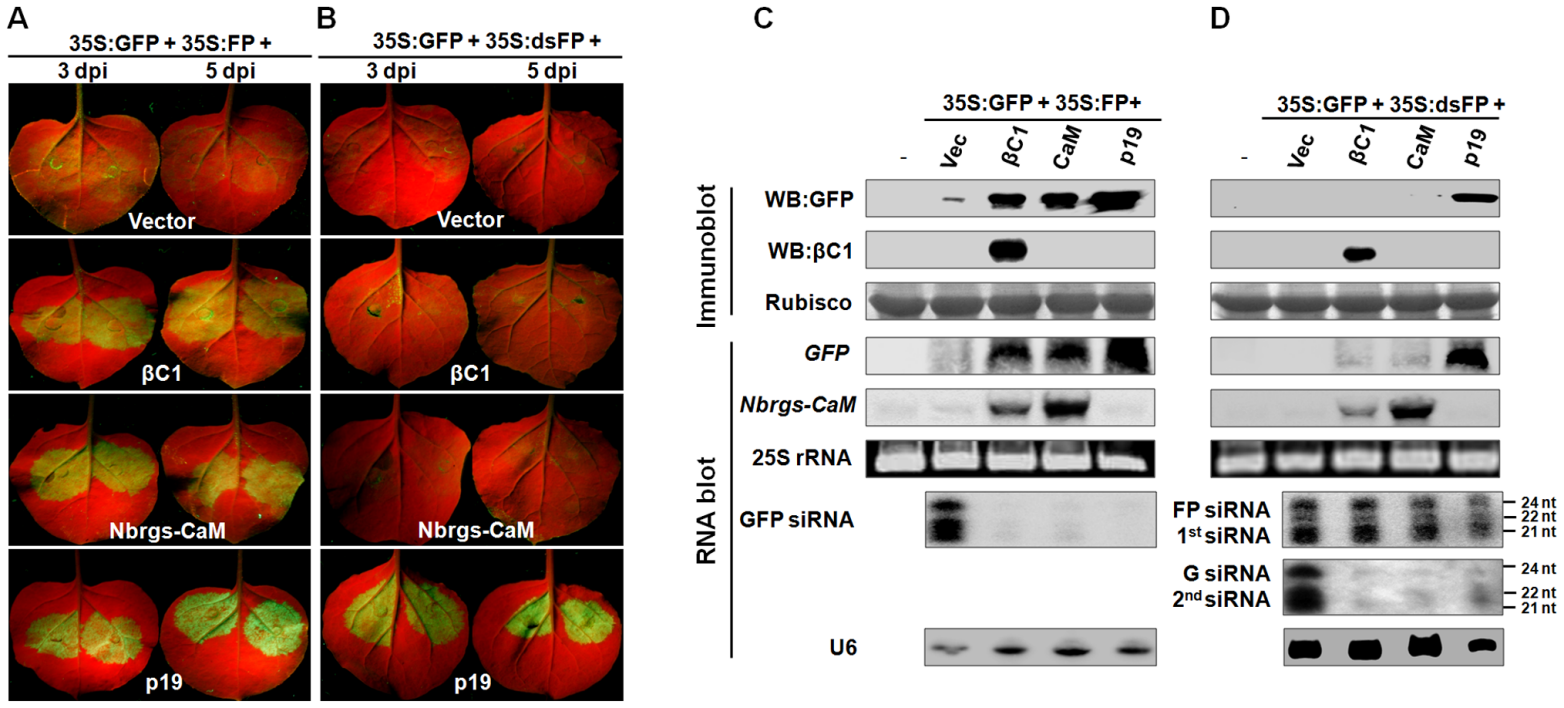
Nbrgs-CaM and βC1 suppress sense-PTGS and the production of secondary siRNA. (**A**) and (**B**) GFP fluorescence in leaves of *Nicotiana benthamiana* plants co-infiltrated *Agrobacterium* cultures expressing the indicated suppressors and GFP reporter (35S:GFP), together with either a sense RNA fragment of GFP (35S:FP) (**A**) or dsRNA fragment of GFP (35S:dsFP) (**B**) as indicated on the top of each panel. The infiltrated leaves were photographed at 3 and 5 dpi under UV light. (**C**) and (**D**) Protein and RNA gel blot analyses of GFP silencing in agroinfiltrated leaf samples shown in (**A**) and (**B**), respectively. GFP or βC1 specific monoclonal antibody was used in immunoblotting. Coomassie blue staining of the large subunit of Rubisco served as loading controls. In the large RNA blots, [α-^32^P]-labeled DNA fragments of *GFP* and *Nbrgs-CaM* were used as probes and ethidium bromide staining of 25S rRNA confirmed the equal loading. For small RNA blots, [γ-^32^P] ATP-labeled GFP oligonucleotides annealed to different region of GFP mRNA were mixed and used as probes in (**C**), whereas labeled GFP oligonucleotides corresponding to “FP” region and “G” regions were used to detect primary FP siRNA (1^st^ siRNA) and secondary G siRNA (2^nd^ siRNA), respectively in (**D**). The U6 RNA blot was used as a loading control of small RNA blot. The sizes of the 21-, 22- and 24-nt RNAs are indicated to the right of the small RNA panel.

In plants, PTGS can be triggered by sense RNA (S-PTGS) or inverted repeat RNA (IR-PTGS). Gene silencing triggered by sense RNA trigger requires the host RNA copy machinery to convert target RNA into dsRNA in the initiation step, whereas inverted repeat RNA or hairpin RNA could directly trigger PTGS [3], [4]. To pinpoint the specific steps targeted by βC1 and Nbrgs-CaM in RNA silencing pathway, we investigated their abilities to suppress S-PTGS and IR-PTGS. For S-PTGS, *N. benthamiana* leaves were co-infiltrated with *Agrobacterium* cultures expressing the suppressors, *GFP* reporter (35S:GFP) and a C-terminal 400 bp-fragment of sense GFP RNA (35S:FP). The procedure of IR-PTGS suppression assay is similar to that of S-PTGS, except that a fragment of GFP dsRNA was expressed from binary vector (35S:dsFP) to serve as a silencing inducer. As shown in Fig. 4A–B, in the absence of a suppressor (vector control), GFP expression was efficiently silenced by simultaneous expression of either the sense RNA trigger (35:FP) or the dsRNA trigger (35S:dsFP) as indicated by red fluorescence in the infiltrated leaves (top row). Similar to the experiments in Fig. 3A, both βC1 and Nbrgs-CaM suppressed GFP silencing triggered by 35S:FP, resulting in bright green fluorescence in co-infiltrated areas of leaves (Fig. 4A, second and third row, respectively). However, neither βC1 nor Nbrgs-CaM suppressed GFP silencing in IR-PTGS, as the infiltrated areas showed red autofluorescence similar to the vector-infiltrated leaves (Fig. 4B, compare second and third rows with top row). As a positive control, p19 suppressed both S-PTGS and IR-PTGS of GFP (Fig. 4A–B, bottom row; Fig. 4C–D), which is consistent with the model that p19 suppresses RNA silencing by sequestering 21-nt siRNA [20]. The GFP imaging data were further confirmed by immunoblot and RNA blot analyses of the accumulation levels of GFP protein and mRNA in infiltrated leaf patches (Fig. 4C–D). The siRNA blots also confirmed that βC1, Nbrgs-CaM and p19 drastically reduced the GFP siRNA during S-PTGS (Fig. 4C). For detection of GFP siRNAs in IR-PTGS assays, we designed two different probes corresponding to the FP portion and G portion of GFP mRNA. The “FP siRNAs” were presumably produced from DCLs-processing of dsRNA precursors transcribed from the 35:dsFP binary vector, and thus represents primary siRNAs. The “G siRNAs” were likely generated from GFP mRNA templates by activities of plant RDRs followed by DCLs-cleavages, and hence should be regarded as secondary siRNAs. Small RNA hybridization showed that both βC1 and Nbrgs-CaM strongly inhibited G siRNA levels without obvious effect on FP siRNAs (Fig. 4D), suggesting that the two proteins suppressed secondary siRNA production. Likewise, Ntrgs-CaM and Slrgs-CaM were also found to suppress S-PTGS but not IR-PTGS of GFP (Fig. S3). Therefore, our data suggest that both βC1 and rgs-CaMs interfere with a step upstream of dsRNA generation in the PTGS pathway.

### Nbrgs-CaM mediates βC1 functions in PTGS suppression and symptom induction

**Figure 5:**
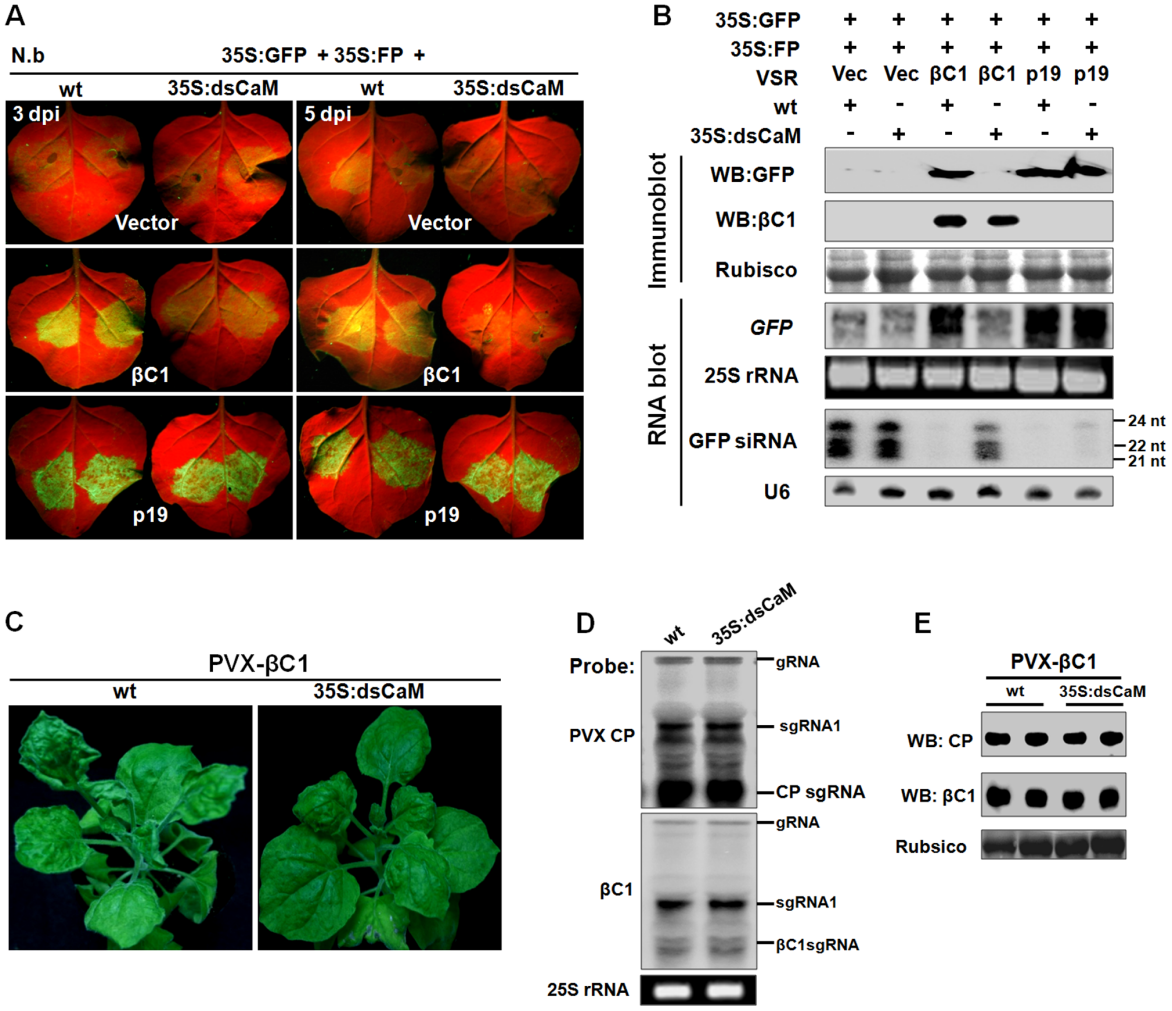
*Nbrgs-CaM* is genetically required for the functions of βC1 in PTGS suppression and symptom modulation. (**A**) GFP fluorescence in leaves of wt and 35S:dsCaM *Nicotiana benthamiana* plants co-infiltrated with 35S:GFP and 35S:FP, together with indicated suppressors. Photographs were taken under UV light at 3 and 5 dpi. (**B**) Protein and RNA gel blot analyses of GFP silencing in agroinfiltrated leaf samples shown in (**A**) at 5 dpi. GFP or βC1 specific monoclonal antibody was used in immunoblotting. Coomassie blue staining of the large subunit of Rubisco served as loading controls. In large RNA blot, [α-^32^P]-labeled DNA fragments of *GFP* and *Nbrgs-CaM* were used as probes and ethidium bromide staining of 25S rRNA confirmed the equal loading. In the small RNA blot, [γ-^32^P] ATP-labeled GFP or U6 oligonucleotides were used as probes. The sizes of the 21-, 22- and 24-nt marker RNAs are indicated to the right of the small RNA panel. (**C**) Symptoms of wt and dsCaM transgenic *N. benthamiana* plants infected with PVX-βC1 at 15 dpi. (**D** and **E**) Analyses of the RNA and protein levels of PVX coat protein (CP) and βC1 in wt and dsCaM transgenic plants infected with PVX-βC1. Total RNAs were extracted from infected plants at 15 dpi and blotted with probes specific for PVX *CP* or *βC1* (**D**), and total protein samples were blotted with specific PVX-CP and βC1 antibodies in a Western blot (**E**). Ethidium bromide staining of 25S rRNA and Coomassie staining are shown to indicate equal loading. The migrations of genomic RNA (gRNA), subgenomic RNA1 (sgRNA1), CP subgenomic RNA (CPsgRNA) and βC1 subgenomic RNA (βC1sgRNA) were indicated.

The up-regulation of *Nbrgs-CaM* by βC1 (Fig. 1), together with their overlapping functions in PTGS suppression and symptom modulation (Fig. 2, 3 and 4), led us to develop a model in which βC1 functions are mediated by Nbrgs-CaM. To test this hypothesis, we first analyzed the ability of βC1 to suppress S-PTGS in wt and 35S:dsCaM plants using a transient agroinfiltration assay. In wt plants, suppression of S-PTGS of GFP by βC1 was confirmed by the presence of strong GFP fluorescence in infiltrated leaf patches under UV light (Fig. 5A), the accumulation of GFP protein and mRNA and the absence of GFP siRNA (Fig. 5B). However, in 35S:dsCaM plants, βC1 failed to suppress GFP silencing (Fig. 5A–B), even though comparable amounts of βC1 were expressed in wt and 35S:dsCaM plants (Fig. 5B). As a positive control, p19 suppressed GFP silencing in both wt and 35S:dsCaM plants (Fig. 5A–B).

To exclude any unspecific defect of the 35S:dsCaM transgenic plants in the βC1 VSR function, we used *N. benthamiana* 16c plants in which the expression of *Nbrgs-CaM* was silenced by the well-characterized *Tobacco rattle virus* (TRV)-based VIGS vector [54], which decreased *Nbrgs-CaM* mRNA levels to 20% of mock-treated plants at 7 dpi (Fig. S4B). Systemically silenced leaves of these plants were then used for *Agrobacterium* co-infiltration in the transient PTGS suppression assay. Again, the abilities of βC1 to suppress GFP silencing and decreased GFP siRNA levels were compromised in *Nbrgs-CaM*-silenced plants, but not in mock-silenced plants (Fig. S4A and S4C). These data collectively suggest that Nbrgs-CaM is required for the ability of βC1 to suppress PTGS and to inhibit siRNA generation.

To further investigate whether βC1-induced developmental abnormities are mediated by Nbrgs-CaM, we infected wt and 35S:dsCaM *N. benthamiana* plants at 6–7 leaf stage with a recombinant PVX vector carrying the *βC1* gene (PVX-βC1). As shown in Fig. 5C, PVX-βC1-infected wt plants developed typical βC1-associated phenotypes such as upward leaf curling and enation, in addition to the PVX mosaic symptoms. However, in PVX-βC1-infected 35S:dsCaM plants, symptoms characteristic of βC1 failed to appear, and only slightly downward leaf curling and mosaic symptoms indicative of PVX infection were observed. Comparable levels of the recombinant PVX genomic and subgenomic RNAs were verified by RNA blot analyses with probes specific to PVX coat protein (CP) and βC1 (Fig. 5D). Furthermore, immunoblotting also confirmed similar expression levels of βC1 and PVX CP in wt and 35S:dsCaM plants (Fig. 5E). Taken together, we conclude that βC1 functions as a VSR and symptom determinant are mediated by Nbrgs-CaM.

### Rgs-CaMs are required for TYLCCNV virulence and genome amplification in *Solanaceae* hosts

**Figure 6:**
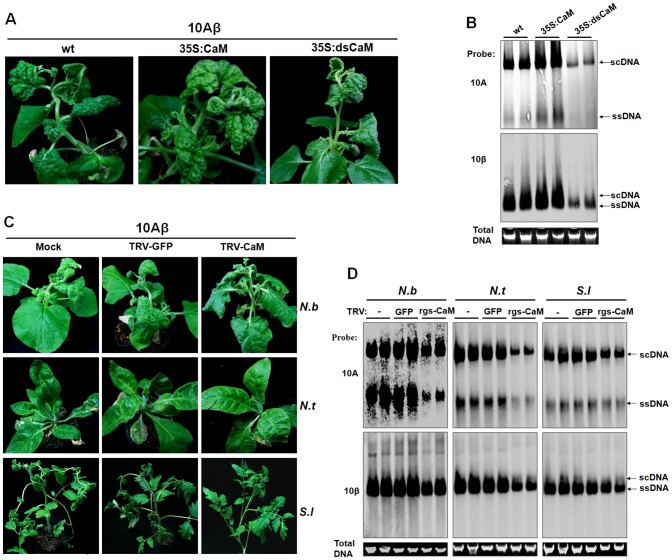
Nbrgs-CaM positively regulated the symptom and accumulation of 10Aβ. (**A**) Symptoms of wild type (wt), overexpression line (35S:CaM) and RNAi line (35S:dsCaM) of Nbrgs-CaM transgenic *Nicotiana benthamiana* plants infected with 10Aβ at 30 dpi. (**B**) Southern blots of 10Aβ accumulation in systemic leaves of wt, 35S:CaM, 35S:dsCaM *N. benthamiana* infected with 10Aβ at 30 dpi. The DNA agarose gel was stained with ethidium bromide as loading control and then blotted using probes specific for 10A and betasatellite (10β). (**C**) Symptoms of rgs-CaM-silenced plants infected by 10Aβ. Partial fragments of *Nbrgs-CaM*, *Ntrgs-CaM* and *Slrgs-CaM* were cloned into the RNA2 of the TRV VIGS vector. *N. benthamiana* (*N.b*), *N. tabacum* (*N.t*), *Solanum lycopersicum* (*S.l*) plants at 4–5 leaf stage were infiltrated with *Agrobacterium* cultures carrying an empty vector (Mock), or pTRV1 with pTRV2-GFP, or with pTRV1 and respective pTRV2-CaM. Rgs-CaM silencing was confirmed in newly emerged leaves 7 days after TRV infiltration by RT-qPCR (Fig. S6). The systemic leaves of these plants were superinfected with 10Aβ and photographs were taken 20 dpi. (**D**) Southern blots of 10Aβ accumulation in systemic leaves of plants shown in (**C**) at 20 dpi. The DNA agrose gel was stained with ethidium bromide as a loading control. Viral single-stranded DNA (ssDNA) and supercoiled DNA (scDNA) are indicated.

Given the important roles of βC1 in TYLCCNV pathogenesis [38], [39], and the involvement of Nbrgs-CaM in these processes (Fig. 5), we anticipated that Nbrgs-CaM has a major function in TYLCCNV infection. To this end, we compared the susceptibilities of *Nbrgs-CaM* transgenic plants and wt plants to 10Aβ infection. After infection, the 35S:CaM plants showed more extensive leaf curling and greater numbers of curled leaves than wt plants. In contrast, the symptoms in infected 35S:dsCaM plants were much milder than those in wt plants (Fig. 6A). The onset of disease symptoms was also advanced by 2 days in 35S:CaM plants (5 dpi compared to 7 dpi in wt plants), but was delayed to 9 to 10 days in 35S:dsCaM plants. Because the #1.1 line of the 35S:CaM plants had only minor abnormality in upper leaves prior to infection (Fig. 2A), the exacerbated symptoms observed in 35S:CaM plants were most likely due to enhanced virulence of 10Aβ in the plants. Accordingly, Southern blot analysis indicated an increased viral DNA accumulations of both helper (10A) and the betasatellite (10β) in 35S:CaM plants. This finding, plus decreased viral DNA accumulations in 35S:dsCaM plants (Fig. 6B), further supports a role of *Nbrgs-CaM* in TYLCCNV infection. We also tested the sensitivities of these *Nbrgs-CaM* transgenic plants to infection with CMV, an unrelated RNA virus. As with the case with 10A and 10Aβ, 35S:CaM plants were more prone to CMV infection whereas 35S:dsCaM plants were more recalcitrant, as judged from symptom severities and viral CP accumulations (Fig. S5). Thus, it appears that *Nbrgs-CaM*-mediated negative regulation of RNA silencing in plants may confer general susceptibilities to virus infections.

Given that expressions of *Ntrgs-CaM* and *Slrgs-CaM* were also induced by TYLCCNB in tobacco and tomato plants, respectively (Fig. S1), and the three rgs-CaMs possessed comparable activity in suppressing PTGS (Fig. 3A–B), we next explored whether Ntrgs-CaM and Slrgs-CaM have similar roles in 10Aβ infection. To this end, *N. benthamiana*, tobacco and tomato seedlings were agro-infiltrated with recombinant TRV vectors which carry partial fragments of *Nbrgs-CaM*, *Ntrgs-CaM* and *Slrgs-CaM*, respectively. Knowdowns of the three *rgs-CaM*s were verified by RT-qPCR, which showed an approximately 80% reduction in their mRNA levels as compared to TRV-GFP-treated plants or mock plants (no TRV infection) at 7 dpi (Fig. S6). *Rgs-CaM*-silenced *N. benthamiana*, tobacco and tomato plants had indistinguishable symptoms from TRV-GFP-infected plants, indicating reductions in the abundance of rgs-CaM do not cause discernible phenotype in these host plants (data not shown). The *rgs-CaM*-silenced plants were infected with 10Aβ and monitored for symptom appearance. As shown in Fig. 6C–D, at 20 days after 10Aβ infection, *rgs-CaM*-silenced plants developed much milder symptoms and accumulated lower amounts viral DNAs than TRV-GFP-treated or mock-silenced plants, suggesting similar roles of these rgs-CaMs in TYLCCNV infection in *Solanaceae* hosts. Overall, our data are consistent with a model that Nbrgs-CaM and its orthologs in *Solanaceae* hosts function as negative cellular regulators of RNA silencing that are induced by βC1 to potentiate TYLCCNV infections.

### Induction of *Nbrgs-CaM* by βC1 represses *NbRDR6* expression

**Figure 7:**
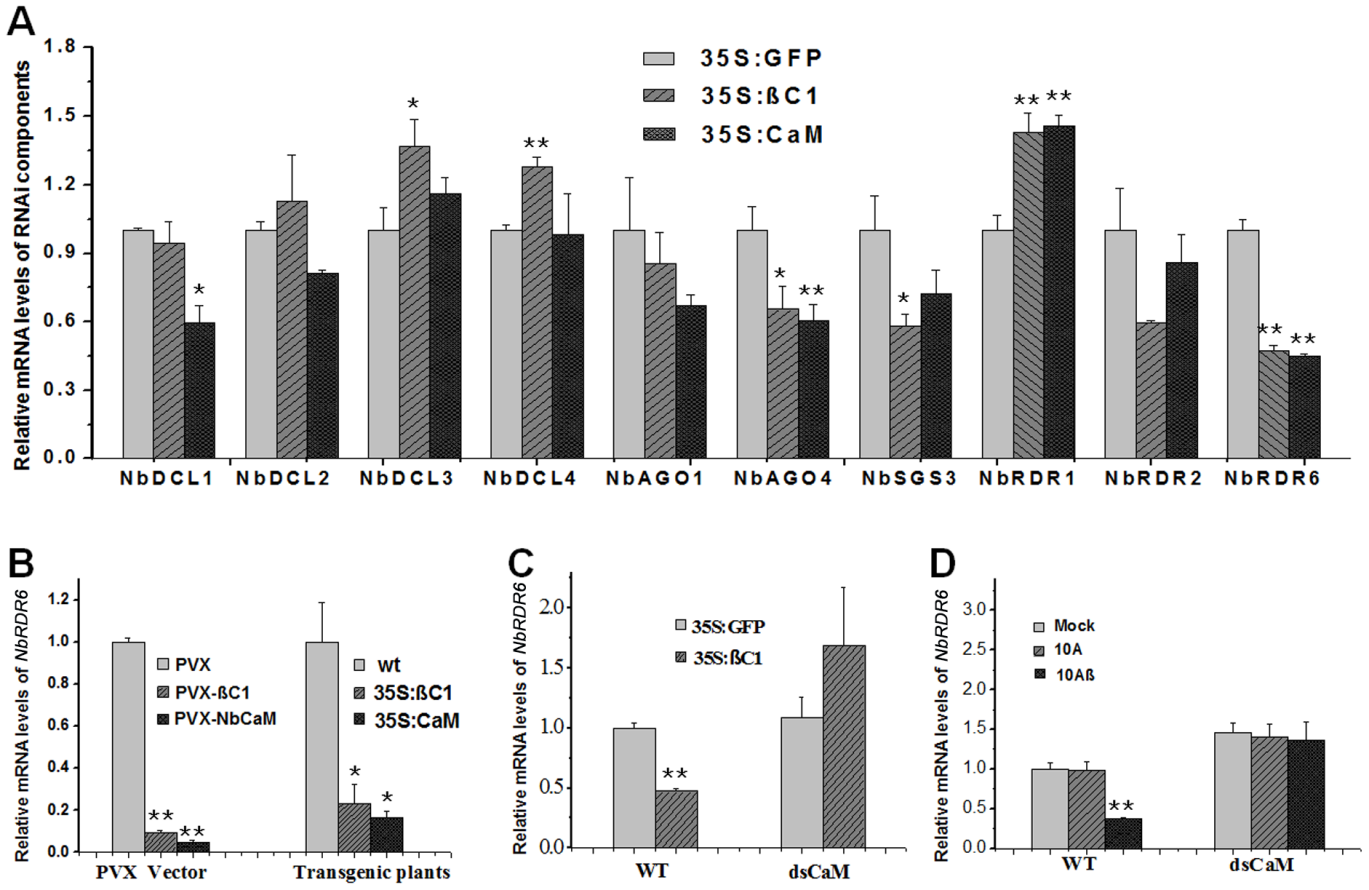
Nbrgs-CaM is genetically required for βC1 to repress *NbRDR6* expression. (**A**) Effects of βC1 and Nbrgs-CaM on the expression of RNAi components. *Nicotiana benthamiana* leaves were infiltrated with *Agrobacterium* cultures expressing 35S:GFP (negative control), 35S:βC1 and 35S: CaM. Total RNAs were extracted from infiltrated zones from three pools of plants at 48 hpi. Relative mRNA levels of *NbDCL1*, *NbDCL2*, *NbDCL3*, *NbDCL4*, *NbAGO1-1*, *NbAGO4-1*, NbSGS3, *NbRDR1, NbRDR2* and *NbRDR6* were analyzed by RT-qPCR with specific primers. The value obtained from 35S:GFP-infiltrated plants was arbitrarily set as 1. (**B**) Levels of *NbRDR6* mRNA in plants infected by a recombinant PVX vector expressing *βC1* (PVX-βC1) and *Nbrgs-CaM* (PVX-NbCaM) versus the vector control (PVX) at 10 dpi (left columns), or in transgenic *N. benthamiana* plants expressing *βC1* and *Nbrgs-CaM* driven by CaMV 35S promoter versus wt plants (right columns). The value in PVX-infected plants or wt plants was arbitrarily set as 1, respectively. (**C**) Levels of *NbRDR6* mRNA in wt and 35S:dsCaM transgenic plants agroinfiltrated with binary plasmids expressing *βC1* and *GFP* at 48 hpi. The value in GFP-infiltrated wt plants was set as 1. (**D**) Levels of *NbRDR6* mRNA in wt and 35S:dsCaM plants infected by TYLCCNV without (10A) or with betasatellite (10Aβ), or mock inoculated plants at 7 dpi. The value in mock plants was set as 1. The mRNA levels of the analyzed genes were normalized to *NbGAPDH* mRNA that served as an internal standard. Each mean value was derived from three independent experiments. Values are means ± SD (*n* = 9). The data were analyzed using Student's *t* test and asterisks denote significant differences between treatments (*P≤0.05, **P≤0.01).

In plants, calmodulins or calmodulin-like proteins regulate a variety of cellular processes by controlling the expression of genes encoding downstream effectors [29], [30]. To investigate whether βC1-induced Nbrgs-CaM altered the expression of components in the RNA silencing pathway, specific primers for RT-qPCR detection were designed to analyze the transcription levels of *N. benthamiana* homologs of *DICER 1* (*NbDCL1*), *DICER 2* (*NbDCL2*), *DICER 3* (*NbDCL3*), *DICER 4* (*NbDCL4*), *ARGONAUTE 1-1* (*NbAGO1-1*), *ARGONAUTE 4-1* (*NbAGO4-1*), *SGS3* (*NbSGS3*), *RDR1* (*NbRDR1*), *RDR2* (*NbRDR2*) and *RDR6* (*NbRDR6*). For this purpose, individual *Agrobacterium* cultures expressing *GFP* (35S:GFP), *Nbrgs-CaM* (35S:CaM) or *βC1* (35S:βC1) were infiltrated into *N. benthamiana* leaves and the transcription levels of RNAi components were measured by RT-qPCR. As compared with the GFP control, expression of *βC1* or *Nbrgs-CaM* at 48 hours post infiltration (hpi) resulted in only moderate changes (60∼150%) in mRNA levels of the RNAi components in repeated experiments (Fig. 7A), although up-regulation of *NbDCL3*, *NbDCL4* and *NbRDR1*, and down-regulation of *NbDCL1*, *NbAGO4* or *NbSGS3* expression by either βC1 or Nbrgs-CaM appeared to be significant (p≤0.05 or p≤0.01). However, the *NbRDR6* mRNA level was consistently reduced by 2-fold by both Nbrgs-CaM and βC1 (Fig. 7A). The inhibitory effect was more evident when *βC1* and *Nbrgs-CaM* were expressed from a PVX vector or from a stable transgene, and in these cases, *NbRDR6* mRNA was reduced to 10∼20% of that of control plants (Fig. 7B). Infections with 10Aβ also decreased the *NbRDR6* mRNA levels to ∼40% of that in 10A-infected or mock-infected plant at 7 dpi (Fig. 7D).

The parallel functions of Nbrgs-CaM and βC1 in PTGS suppression and *NbRDR6* down-regulation led us to postulate that Nbrgs-CaM is required for βC1 to repress *NbRDR6* expression. To test this possibility, *Agrobacterium* cultures harboring expression cassettes of the *GFP* (control) or *βC1* were infiltrated into leaves of wt and 35S:dsCaM plants, and the effects on *NbRDR6* transcription were measured by RT-qPCR. As compared to expression of *GFP*, expression of *βC1* reduced the *NbRDR6* mRNA levels by more than 2-fold in wt plants. However, upon transient expression of *GFP* and *βC1* in 35S:dsCaM plants, the *NbRDR6* mRNA levels were comparable (Fig. 7C). Likewise, 10Aβ infection also led to reduced *NbRDR6* expression in wt plants but not in in 35S:dsCaM plants, when compared to that of 10A-infected or mock plants (Fig. 7D). These results indicate that the requirement of *Nbrgs-CaM* for repression of *NbRDR6* transcription is likely to be biologically relevant. It is worth noting that higher basal levels of *NbRDR6* mRNA (∼1.5-fold) were observed in 35S:dsCaM plants than in wt plants (Fig. 7D, compare mock-treatment in dsCaM and wt plants), suggesting that endogenous Nbrgs-CaM suppresses *NbRDR6* expression. Taken together, our data suggest that Nbrgs-CaM acts downstream of βC1 to suppress *NbRDR6* expression.

### RDR6 mediates RNA silencing-based antiviral defense against TYLCCNV infection in *N. benthamiana* and *Arabidopsis*

**Figure 8:**
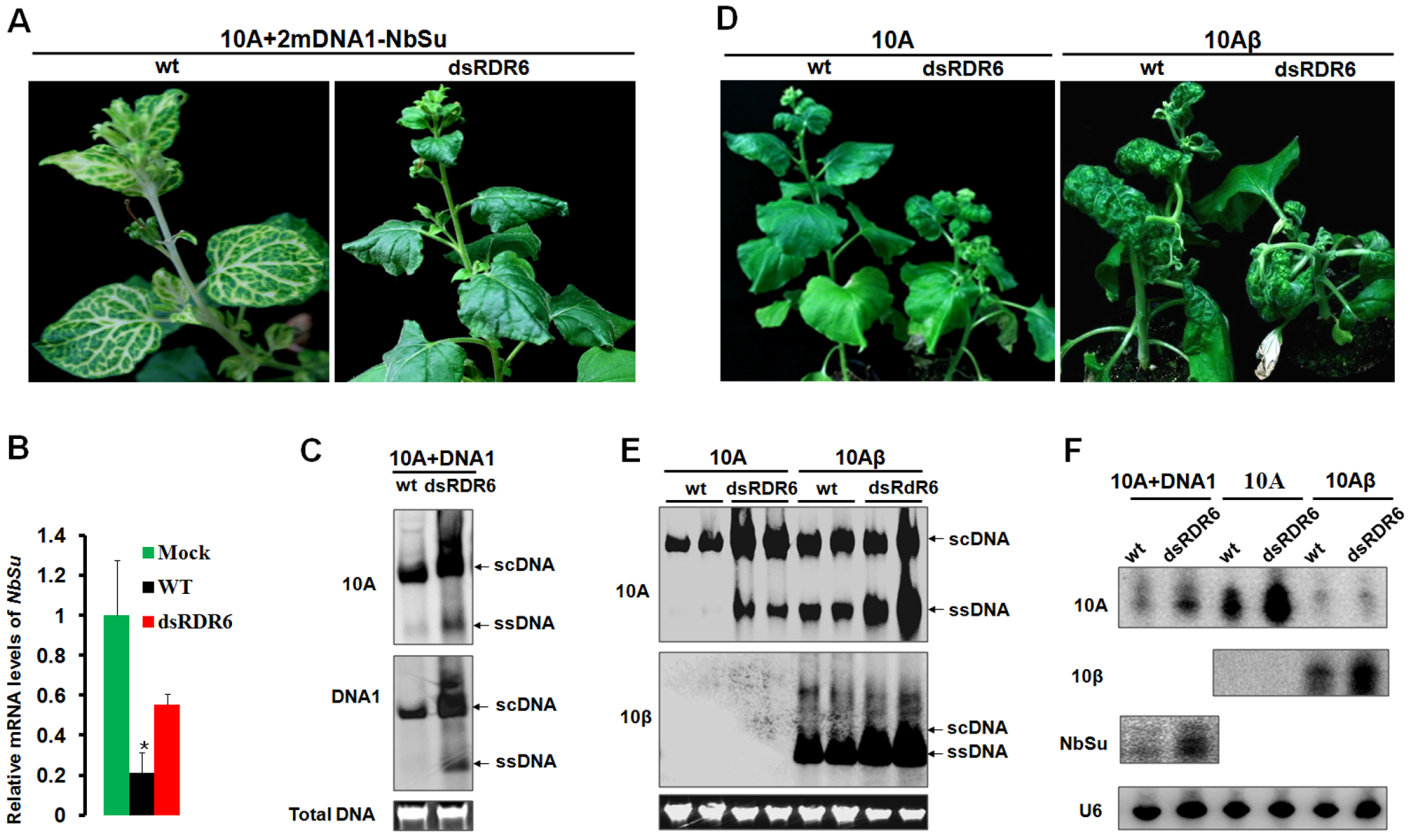
Compromised VIGS efficiency and enhanced susceptibility to TYLCCNV infection in RDR6-deficient *Nicotiana benthamiana* plants. (**A**) Phenotype of *Su* silencing in the wt and *NbRDR6* RNAi line (dsRDR6) induced by the 10A+2mDNA1-*NbSu* VIGS vector at 30 dpi. (**B**) RT-qPCR analysis of levels of *Su* mRNA in silenced plants shown in (**A**). Mock indicates wt plants infected by the empty VIGS vector (10A+2mDNA1) and the relative *Su* mRNA value in mock plants was set as 1. Error bars: ± SD. Asterisk indicates P value compared with mock-treated wild type plants: *P≤0.05 (Student's *t* test). (**C**) Southern blot analysis of 10A and DNA1 accumulation in systemic leaves of *Su*-silenced plants shown in (**A**) at 30 dpi. (**D**) Symptoms of wt and dsRDR6 *N. benthamiana* plants infected with 10A or 10Aβ at 30 dpi. (**E**) Southern blot analysis of viral DNA accumulation in systemically infected leaves of wt and dsRDR6 at 30 dpi shown in (**D**). Total DNA agarose gels were stained with ethidium bromide to show the equal loading. Viral single-stranded DNA (ssDNA) and supercoiled DNA (scDNA) forms are indicated. (**F**) RNA blot hybridization analysis of viral small RNA accumulations in VIGS vector-infected plants shown in (**A**) and wt virus-infected plants shown in (**D**). The blots were hybridized to single-stranded DNA oligonucleotide probes specific for 10A, 10β, *NuSu* and U6 small RNA.

To verify the role of NbRDR6 in host antiviral defense against TYLCCNV infection, we used a *N. benthamiana NbRDR6* RNAi line (dsRDR6) described earlier [13]. We first compared the efficiency of *Su* silencing induced by the 10A-derived VIGS vector in wt and dsRDR6 *N. benthamiana* plants. At 30 dpi, typical *Su* silencing phenotypes were developed on wt plants, but not on dsRDR6 plants (Fig. 8A). RT-qPCR analysis revealed a 5-fold reduction of *Su* mRNA in wt plants compared to mock-silenced plants (infected by 10A+2mDNA1), whereas in dsRDR6 plants *Su* mRNA was reduced less than 2-fold (Fig. 8B), confirming the defective *Su* silencing in dsRDR6 plants (Fig. 8A). Interestingly, in dsRDR6 plants, both DNA components of the viral vector accumulated to higher levels than in wt plants (Fig. 8C), suggesting that the compromised *Su* silencing in dsRDR6 plants is due to an inability to mount RNA silencing response upon TYLCCNV infection, rather than a failure to support robust virus replication.

The functions of NbRDR6 in RNA silencing-based defense were further tested by infection of 10A and 10Aβ. As described in earlier studies [38], infection of wt plants with 10A elicited very mild symptoms, which were greatly exacerbated when the pathogenesis-enhancing satellite was present (10Aβ) (Fig. 8D). Interestingly, in dsRDR6 plants, infection with the 10A helper alone resulted in severe downward leaf curling. However, the phenotypes characteristic of the betasatellite infection, including shoot bending, leaf distortion, vein thickening and enations, were not observed (Fig. 8D). Infection of dsRDR6 plants with 10Aβ produced slightly severer symptoms than those in wt plants (Fig. 8D), and the disease onset was also advanced in dsRDR6 plants (3∼4 dpi) as compared to those in wt plants (6∼7 dpi). Southern blot hybridization analysis of 10A-infections also revealed that total viral DNA accumulated at higher levels in dsRDR6 plants than in wt plants (Fig. 8E). Similarly, betasatellite co-infections also resulted in increased helper viral DNA accumulation. In particularly, the single-stranded viral DNA forms (ssDNA) were barely visible in 10A-infected wt plants, but were greatly enhanced in dsRDR6 plants and in betasatellite co-infected plants (Fig. 8E). Previously, it was thought that disease phenotypes induced by 10Aβ could be solely attributed to the expression of the pathogenesis factor βC1, because infections with 10A alone elicited negligible symptoms (Fig. 8D). However, our data here suggest that betasatellite-mediated amplification of 10A, likely as a result of repression of *NbRDR6*, also contributes to disease manifestation. Small RNA blot analyses also showed that betasatellite suppressed viral siRNA (vsiRNA) production (Fig. 8F, compare 10Aβ-infectd plants with 10A-infected plants). Interestingly, vsiRNAs accumulated at higher levels in dsRDR6 plants when compared with wt plants, regardless of the presence or absence of the betasatellite, so is also the case with the *NbSu*-derived siRNAs (Fig. 8F). Nevertheless, the abundant siRNAs observed in dsRDR6 plants apparently failed to efficiently silence their targets (Fig. 8A–C) or to confer effective antiviral defense (Fig. 8D–E).

**Figure 9:**
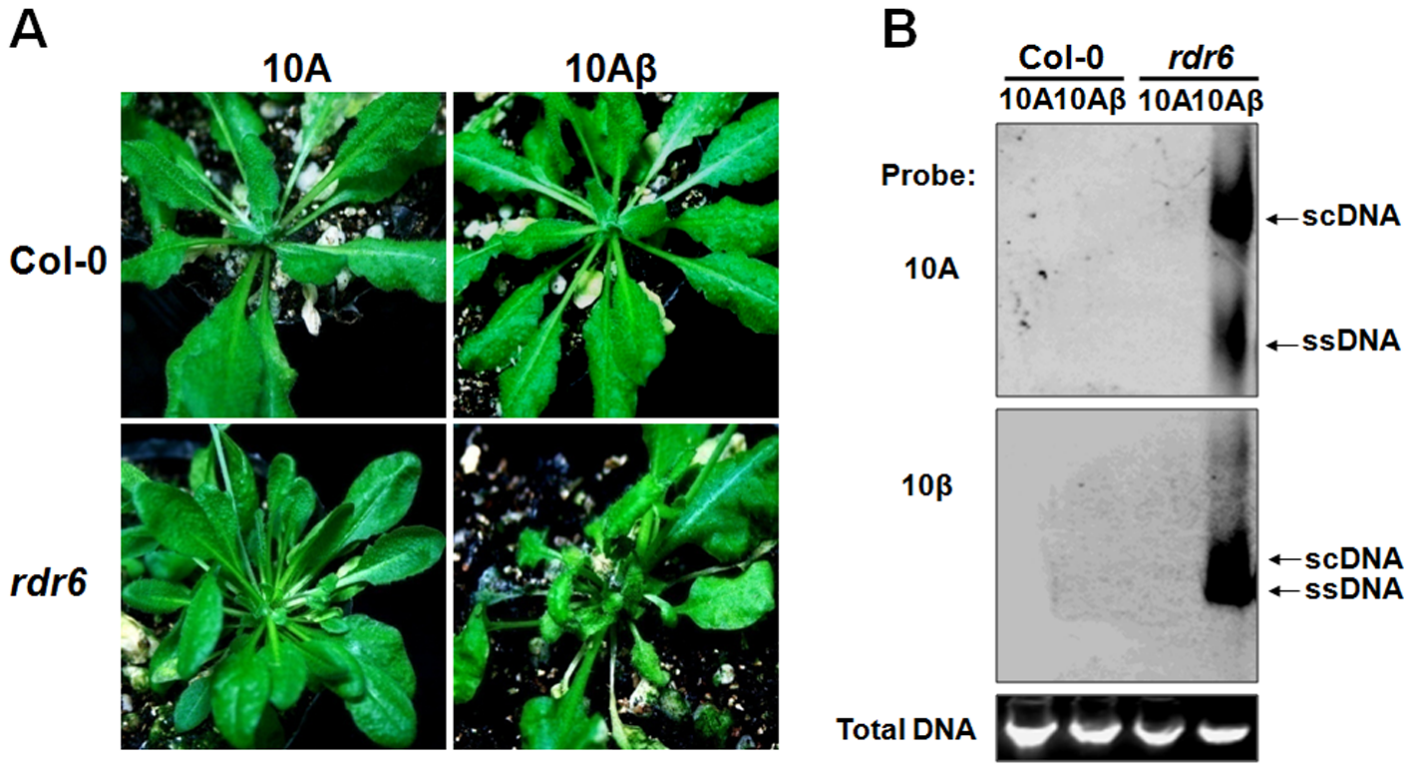
RDR6 mediates nonhost defense against TYLCCNV in *Arabidopsis*. (**A**) Symptoms of wt (Col-0) and *rdr6-11 Arabidopsis* plants infected by 10A and 10Aβ at 30 dpi. (**B**) Southern blot analysis of viral DNAs of 10A and 10β in systemically infected leaves of Col-0 and *rdr6-11* plants at 30 dpi. The total DNA agarose gel was stained with ethidium bromide to show the equal loading. Viral single-stranded DNA (ssDNA) and supercoiled DNA (scDNA) forms are indicated.

*A. thaliana* is known as a susceptible host for only a few geminiviruses including CaLCuV and *Beet curly top virus*. TYLCCNV, either alone (10A) or in association with betasatellite (10Aβ), has consistently failed to systemically infect *Arabidopsis* ecotype Columbia (Col-0) (Fig. 9 A–B). Remarkably, *Arabidopsis rdr6* mutant plants were susceptible to infection of 10Aβ, showing typical downward leaf curling on new leaves starting at 7 dpi (Fig. 9A). The narrow curled leaves and cotyledons of infected plants were reminiscent of the phenotypes induced by transgenic expression of *βC1* in *Arabidopsis* [55]. After 30 dpi, the infected plants began to develop necrosis on newly expanded leaves and shoots, and some of the shoots eventually died. However, in contrast to 10Aβ, 10A alone failed to systemically infect *rdr6* plants (Fig. 9A). Consistent with the observed symptoms, Southern blot analysis showed high levels of helper and satellite DNA accumulations in leaves of *rdr6* plants systemically infected with 10Aβ, whereas viral DNAs were absent in inoculated wt plants or 10A-inoculated *rdr6* plants (Fig. 9B). These data strongly suggest a major role of RDR6 in host antiviral defense against TYLCCNV infection and underscore the function of RDR6-mediated RNA silencing in restricting the host range of a geminivirus.

## Discussion

In plants, RNA silencing represents a major defense mechanism against virus infection. Consequently, plant viruses have evolved to encode VSRs as potent molecular arms to counteract antiviral RNA silencing [1], [2]. These structurally distinct VSRs often interact with various components in the RNA silencing pathway, including long or short dsRNA duplex, AGOs, DCLs and RDRs as well as their functional partners, thereby to disable the RNA silencing-based host defense systems [15], [16]. Besides an antiviral role, RNA silencing also regulates essential developmental processes such as endogenous gene expression and genome stability [31]. Given the important role of RNA silencing in normal cell physiology and its “spreading” nature by means of signal amplification, it is not surprising that this process requires appropriate controls by cellular regulatory factors or pathways. Indeed, genetic screenings have identified several endogenous silencing suppressors or anti-silencing factors involved in the negative regulation of RNA silencing [56]–[60]. These factors may function to reactivate the expression of certain silenced genes or to prevent undesirable action of RNA silencing. It is conceivable that those endogenous pathways may be explored by gene-poor viruses to counterattack host antiviral responses. Such a scenario has been suggested by the identification of the calmodulin-like protein Ntrgs-CaM in tobacco, which interacts with and is induced by the TEV-encoded VSR HC-Pro [27]. However, it is not known whether Ntrgs-CaM is required for HC-Pro VSR functions. Here we report that a DNA geminivirus-encoded βC1 protein up-regulates the endogenous *Nbrgs-CaM* gene and its orthologs in *Solanaceae* hosts. Furthermore, PTGS suppression and symptom induction by βC1 are mediated by induction of *Nbrgs-CaM* and likely by subsequent repression of RDR6-mediated antiviral RNA silencing defense responses. The identification of rgs-CaM as a common factor for two unrelated VSRs suggests that subversion of ESR may be a common and effective strategy whereby viruses to suppress RNA silencing.

It has previously been shown that the Ntrgs-CaM suppresses VIGS and reverses established PTGS, thus representing the first identified ESR [27]. Recently, however, an opposite effect of tobacco rgs-CaM on VSRs has been reported by Nakahara and co-workers, leading to the proposal that tobacco rgs-CaM acts as a host defense measure by interacting with and quenching VSRs that contain dsRNA binding domain [61]. Nakahara et al. have also showed that transgenic tobacco plants over-expressing rgs-CaM promote the degradation of VSRs, including HC-Pro of potyviruses and 2b of cucumoviruses, and thus are less susceptible to infections of these viruses [61]. However, here our data are not in agreement with the proposed function of rgs-CaM as a counter-VSR factor, but instead reinforce the notion that rgs-CaMs are *bona fide* ESRs based on the following observations: (i) rgs-CaMs suppress S-PTGS and secondary siRNA biogenesis in several transient assays based on conventional agroinfiltration (Fig. 3A–B, Fig. S3 and Fig. 4); (ii) transgenic over-expression of *Nbrgs-CaM* suppresses VIGS of an endogenous gene whereas down-regulation of *Nbrgs-CaM* enhances silencing phenotypes (Fig. 3C, Fig. S2); (iii) over-expressed *Nbrgs-CaM* induces phenotypes resembling those of VSR-expressing plants (Fig. 2A); (iv) Nbrgs-CaM suppresses *NbRDR6* expression (Fig. 7) and consequently, down-regulation of *NbRDR6* suppresses geminivirus-VIGS (Fig. 8A); (v) leaves of both 35S:CaM and dsRDR6 *N. benthamiana* plants inhibit S-PTGS but not IR-PTGS in an agroinfiltration assay (Fig. S7); (vi) finally, 35S:CaM plants have enhanced susceptibility to TYLCCNV and CMV infections whereas 35S:dsCaM plants are more resistant (Fig. 6, Fig. S5). We also did not observe changes in the stability of βC1 or CMV 2b in plants with altered *Nbrgs-CaM* mRNA levels (Fig. 5, Fig. S5), suggesting that Nbrgs-CaM do not direct these VSRs for degradation. In addition, we provide genetic evidence that Nbrgs-CaM mediated the βC1 functions in PTGS suppression and symptom induction (Fig. 5). Our findings have thus revealed a positive role for Nbrgs-CaM in VSR function and virus infection. These seemingly contradictory findings could be due to different experimental conditions employed, since Nakahara et al. also determined that Ntrgs-CaM suppresses RNAi when expressed in *Drosophila* S2 cells [61]. Alternatively, it is possible that rgs-CaM homeostasis contributes to the proper regulation of RNA silencing. In support of the latter hypothesis, Roth et al. observed that only moderate levels of Ntrgs-CaM suppress sense-transgene silencing when ectopically expressed in *Arabidopsis*, whereas highly expressed Ntrgs-CaM does not [62]. This suggests that the suppression activity of Ntrgs-CaM may be subjected to negative feedback regulation. Although several *Nbrgs-CaM* transgenic lines generated in our study displayed uniform activity in VIGS suppression (Fig. S2), we cannot exclude the possibility that a certain range of Nbrgs-CaM expression levels might have different effect.

Unlike HC-Pro, which is a cytoplasmic protein and binds to siRNA duplex [63], βC1 of TYLCCNB primarily localizes in nuclei and binds to single- but not double-stranded RNA [41], [64]. It is unclear how structurally unrelated HC-Pro and βC1 have evolved independently to induce *rgs-CaM*. Induction of *rgs-CaM* was not observed for p19 of TBSV (Fig. 3B), another well-characterized VSR with a siRNA-binding domain [20], suggesting that exploitation of rgs-CaM is not a common feature associated with VSRs. A recent study has shown that an ethylene inducible RAV/EDF transcription factor is required for the HC-Pro functions as a VSR and symptom modulator [28]. Subsequent microarray analyses have revealed differential expression of many biotic and abiotic stress response genes in *Arabidopsis* in response to HC-Pro in a *RAV2*-dependent manner. Among these are *Arabidopsis* FIERY1 (AtFRY1), which negatively regulates transitive silencing [57], and AtCML38 (Calmodulin-like protein 38), a closely related homolog of rgs-CaM in *Arabidopsis* (Fig. 1D), both of which were up-regulated [28]. Interestingly, our global gene expression analysis also revealed that many ethylene responsive transcription factors and stress-related genes are differentially up-regulated in *βC1* transgenic plants. Notably, the list includes the *N. benthamiana* homolog of *RAV2* (*NbRAV2*) (unpublished data). It has been shown that biotic and abiotic stresses, or treatment with Ethephon, a synthetic compound which decomposes into ethylene, could divert plants from antiviral silencing to cope with other stress responses [65]. Thus, it is tempting to speculate that HC-Pro and βC1 may have convergently evolved a mechanism for PTGS suppression through induction of the ethylene-mediated stress response. Further studies are needed to determine whether an *N. benthamiana* ortholog of RAV2 mediates the induction of *Nbrgs-CaM* by βC1, and whether AtFRY1 and AtCML38 are downstream mediators of HC-Pro. Alternatively, βC1 has also been shown to be a TGS suppressor and when ectopically expressed, βC1 causes global reductions in host genome cytosine methylation [66]. It is possible that the transcription of Nbrgs-CaM is silenced by a DNA methylation-related mechanism under normal conditions, which is derepressed by the TGS suppressor function of βC1.

Our data reveal that up-regulation of *Nbrgs-CaM* expression by βC1 represses *NbRDR6* expression (Fig. 7). Consistence with this finding, both βC1 and rgs-CaMs suppress S-PTGS but not IR-PTGS, and inhibits the production of secondary siRNAs (Fig. 4, Fig. 8F and Fig. S3), suggesting that βC1 and rgs-CaMs act to inhibit dsRNA formation catalyzed by the activity of cellular RDRs. This notion is further reinforced by the observation that both 35S:CaM and dsRDR6 plants are defective in S-PTGS but not IR-PTGS in a conventional agroinfiltration assay (Fig. S7). Perhaps the most convincing genetic evidence to demonstrate a specific role for VSRs is rescue of the infections of VSR-deficient mutant viruses with host cells defective in RNA silencing [67]. Such an approach has been used to establish the antiviral role of RDRs-mediated secondary vsiRNAs biogenesis against the infections of several plant RNA viruses [6], [9], [10], and to reveal the natural antiviral functions of RNAi in mammals [68], [69]. It has been shown that 10A and some other monopartite begomoviruses are incapable of blocking host RNA silencing and inducing typical symptoms, and that their betasatellites are required for the full virulence of the helper viruses [43], [44]. Here our data showed that the 10A DNA accumulation and pathogenicity can be partially rescued in *N. benthamiana* plants deficient in *NbRDR6* (Fig. 8D–E), underscoring the specific roles of βC1 in suppressing RDR6 functions. The essential role of RDR6 in antiviral defense has been reinforced by our observations that *Arabidopsis rdr6* mutant plants were susceptible to 10Aβ, which otherwise can not establish robust infection in wt plants (Fig. 9). Previously, it has been reported that AGO2-mediated RNA silencing defense confers nonhost resistance to PVX infection of *Arabidopsis* [70]. To our knowledge, our data represent the first evidence that RNA silencing functions to constrain the host range of a DNA virus.

**Figure 10:**
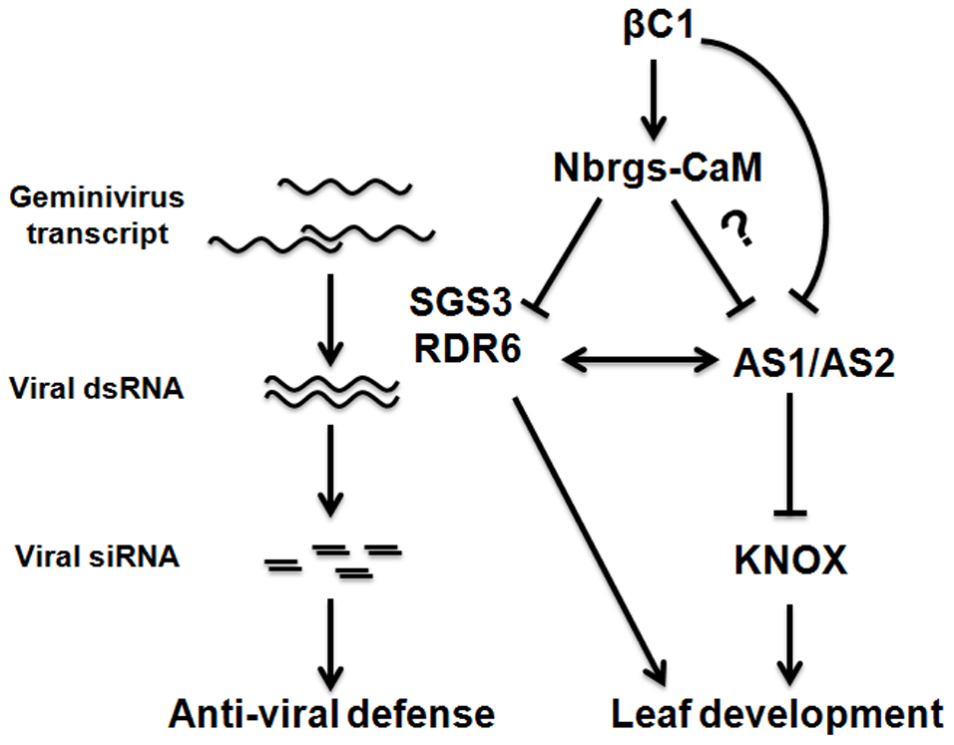
A working model to explain the roles of the endogenous suppressor Nbrgs-CaM in counterdefense and symptom induction by TYLCCNB βC1. Nbrgs-CaM is a cellular negative regulator of RNA silencing, which represses the expression of *RDR6*. RDR6, together with SGS3, presumably monitors geminivirus-derived transcripts and subsequently converted them into dsRNAs that trigger RNA silencing. βC1 encoded by TYLCCNB up-regulates *Nbrgs-CaM* to repress *RDR6* expression, thereby counterattacking host RNA silencing. Nbrgs-CaM is also required for βC1-induced symptom defects. βC1 directly interacts with ASYMMETRIC LEAVES1 (AS1) (67), or may indirectly regulate AS1/AS2 (via Nbrgs-CaM and/or NbRDR6), to induce developmental defects.

RDR6 was originally identified in *Arabidopsis* as required for PTGS triggered by sense-transgenes (S-PTGS) but not by inverted repeat RNA (IR-PTGS) or by RNA viruses such as PVX (RNA-VIGS) [3]. Unlike RNA viruses, geminiviruses lack a dsRNA phase in their life cycle and thus do not obligatorily trigger RNA silencing. It has been suggested that abundant geminiviral transcripts could be perceived as aberrant RNAs and subsequently be recruited by host RDRs as templates to produce dsRNA [30]. In this sense, geminivirus-induced gene silencing is similar to S-PTGS in the initial stage because both processes require the actions of host RDRs (Fig. 10). In support of this notion, VIGS of an endogenous gene in *Arabidopsis* by CaLCuV-derived geminiviral vector requires RDR6 as well as SGS3 [33]. Our data also shows that VIGS of the *Su* gene by the 10A-derived vector was compromised in *NbRDR6*-deficient *N. benthamiana* plants (Fig. 8A–B). Another hypothetic origin for geminiviral dsRNA precursor are the 3′ end overlapping transcripts generated by convergent transcription on the viral circular genome, which could be DCLs substrates for generation of vsiRNA (Fig. 10). Notably, SGS3, the dsRNA binding partner of RDR6, specifically recognizes dsRNAs with 5′ overhangs, a structure analog to 3′ end partially overlapped geminiviral transcripts [71]. This raises the intriguing possibility that such viral transcripts or their derivatives could be recruited by SGS3 and RDR6 to produce dsRNA precursors for vsiRNAs. Interestingly, TYLCV V2 suppresses PTGS likely through interacting with SGS3 and/or competing for binding to dsRNA substrates [71], [72]. As with TYLCCNV βC1, TYLCV V2 also suppresses S-PTGS but not IR-PTGS [73], indicating that interference with SGS3/RDR6-mediated dsRNA formation is a common theme for geminiviruses-encoded VSRs. Interestingly, Aregger et al. have recently found that CaLCuV-derived siRNA populations are largely unaffected by *Arabidopsis rdr1/2/6* triple mutation using deep sequencing and blot hybridization [74], suggesting that the bulk of vsiRNAs are RDR1, 2 and 6-independent (i.e. primary vsiRNAs). It is worth mentioning that in addition to RDR1, 2 and 6, the *Arabidopsis* genome also encodes three poorly characterized RDRs of the γ class namely RDR3, RDR4 and RDR5. The prevailing assumption is that these three RDRs play no major role in antiviral defense. However, a recently study report that the tomato yellow leaf curl geminivirus *Ty-1* and *Ty-3* resistance genes code for a γ Class RDR that represents tomato homologs of *Arabidopsis* RDR3/4/5 [75], suggesting that this group of RDRs in *Arabidopsis* may also be involved in vsiRNA biogenesis. Here we have shown that after 10A infection (either alone, with betasatellite or with DNA1 component), even higher levels of vsiRNAs accumulated in *N. benthamiana* dsRDR6 plants than in wt plants (Fig. 8F), and vsiRNA levels are proportional to the viral DNA accumulations in these plants. Therefore, these abundant RDR6-independent vsiRNAs (assuming that the residual RDR6 activity is negligible in dsRDR6 plants) seem to be unable to silence their targets efficiently, as manifested by the compromised *NuSu* VIGS (Fig. 8A–C) and by the hypersensitivity to 10A infections in the dsRDR6 plants (Fig. 8D–E). It appears that plants defective in *RDR6* are unable to mount an effective RNA silencing response upon TYLCCNV infections. As a result, TYLCCNV multiplies to higher levels, which, in turn, may produce more aberrant mRNA transcripts to generate abundant primary vsiRNAs or secondary vsiRNAs via the activities of other RDRs. It has been suggested that *Arabidopsis* RDR6 functions as a genome surveillance factor to monitor aberrant mRNAs derived from transgenes and targets those mRNAs for PTGS [76]. Given the resemblance of transgene-derived mRNAs and abundant geminiviral transcripts, such an RDR6-mediated protection mechanism may also operate to detect geminivirus infections. Alternatively, RDR6 may act at systemic levels to potentiate distal tissues to initiate an immediate early response against virus infection. Previously, Schwach and associates have shown that RDR6 is not required for production of PVX-derived siRNAs, but prevents systemic PVX infection likely through amplification of systemically movable silencing signals [13]. It remains to be determined whether RDR6 function at cellular level or systemic tissue, or both, to defend against geminivirus infections.

It is important to note that the betasatellite greatly suppresses vsiRNA biogenesis in infected wt and dsRDR6 plants (Fig. 8F), which is consistent with its activities on GFP siRNA production observed in agroinfiltration assays (Fig. 3B, Fig. 4C–D, Fig. 5B and Fig. S3C–D). However, the dominant effect of TYLCCNB βC1 on vsiRNA biogenesis can not be solely attributed to its function in *RDR6* suppression, because knockdowns of *NbRDR6* do not reduced vsiRNA production (Fig. 8F). Therefore, expression of *βC1* likely has pleiotropic effects on the host RNA silencing pathway. Indeed, transient expression of *βC1* also reduced the mRNA levels of *NbSGS3* and *NbAGO4* (P≤0.05), and in this regard, *NbAGO4* is also suppressed by Nbrgs-CaM (P≤0.01) (Fig. 7A). Nuclear-replicating geminiviruses encounter host RNA silencing defense at both transcriptional (TGS) and post-transcriptional (PTGS) levels [33], [34]. Raja and associates have shown that the *Arabidopsis* homolog of NbAGO4 plays an important role in methylation-based epigenetic defense against geminivirus [77]. In addition, we have recently reported that TYLCCNB βC1 suppresses TGS through interactions that inhibited the activity of S-adenosyl homocysteine hydrolase (SAHH), a key enzyme in the host DNA methylation pathway [66]. Notably, the fact that 10A alone is not able to infect *Arabidopsis rdr6* mutant suggests that suppression of other layers of host defense by betasatellite are required for 10A to establish robust infection in this host species.

Another interesting aspect of our finding is the similar phenotypes of 35S:CaM and 35S:βC1 plants, and the genetic requirement of *Nbrgs-CaM* for βC1-disease manifestation (Fig. 5A–B). Down-regulation of *NbRDR6* alone by Nbrgs-CaM cannot account for the observed phenotypic defects observed in βC1 and 35S:CaM plants, since dsRDR6 plants have largely normal phenotypes (data not shown; also in [12]–[13]). Therefore, other unknown targets downstream of Nbrgs-CaM may be involved in the development of βC1-induced symptoms. The upward-curled leaf phenotypes in βC1 and 35S:CaM plants suggest that these proteins may disrupt the leaf adaxial–abaxial identity. In *Arabidopsis*, ASYMMETRIC LEAVES1 (AS1) and AS2 are important factors for promoting the establishment of leaf adaxial-abaxial identity. AS1 and AS2 form a repressor complex that binds directly to the regulatory motifs present in promoters of the *KNOX* genes that specify leaf polarity [78]. It has been shown that *Arabidopsis* RDR6, as well as SGS3 and AGO7 in the trans-acting siRNA pathway, genetically interact with AS1/AS2 to synergistically regulate leaf development. The *rdr6* and *as1 or as2* double mutants display an abnormal leaf adaxial identity with a ruffled surface [79]–[80] that is reminiscent of the phenotypes shown in βC1 and 35S:CaM transgenic plants. Interestingly, Han et al. have recently showed that *Arabidopsis* calmodulin physically interacts with AS1 and relieves the AS1/AS2-suppression of *KNOX* transcription [81]. It remains to be determined whether Nbrgs-CaM has a similar effect in *N. benthamiana*. Yang et al. have also suggested that TYLCCNB-encoded βC1 uses molecular mimicking of AS2 to form a complex with AS1 to regulate leaf development [55]. Therefore, multiple pathways involved in leaf development may be affected by the βC1, which could lead to disease manifestations (Fig. 10). Future studies are needed to address the effects of βC1 on the transcription levels of specific downstream targets of the AS1-AS2 pathway and the RDR6-SGS3-AGO7 pathway.

## Materials and Methods

### Plant materials and growth conditions

*N. benthamiana* seedlings were potted in soil and placed in an insect-free growth chamber at 25°C and 60% relative humidity under a 16 h light/8 h dark photoperiod. 35:βC1 transgenic *N. benthamiana* lines were generated in a previous study [36], and the transgenic GFP 16c and dsRDR6 lines were generous gifts of David C. Baulcombe. The *A. thaliana* ecotype Columbia (Col-0) and *rdr6-11* mutant were used for this study. Seeds were surface sterilized with 75% ethanol and 50% bleach, and then washed three times with sterile water. Sterile seeds were suspended in 0.05% agarose and plated on Murashige and Skoog (Duchefa Biochemie, Haarlem, Netherlands) medium plus 2.0% sucrose. Plates were stratified in darkness for 3 d at 4°C and then transferred to a tissue culture room at 22°C under an 8-h-light/16-h-dark photoperiod. After 2 weeks, seedlings were potted in soil and placed in a growth chamber at 22°C and with 70% relative humidity under an 8-h-light/16-h-dark photoperiod.

### Cloning full-length rgs-CaM cDNAs and plasmid constructs

The tobacco rgs-CaM sequence was used to identify orthologous sequences from available *N. benthamiana* ESTs. BLAST searches revealed high homology between rgs-CaM from tobacco and *N. benthamiana*. Primers designed to anneal to conserved sequences in the 5′ and 3′ untranslated regions of tobacco rgs-CaM were used to amplify the coding region of *N. benthamiana* rgs-CaM by reverse transcription PCR (RT-PCR). Amplification with primer pairs CaM-cds-F/CaM-cds-R yielded a specific product of approximately 600-bp, which was cloned into pMD18-T (TaKaRa, Dalian, China) and sequenced. Detailed primer information is listed in Table S1. The full-length coding sequence of *Nbrgs-CaM* was deposited in GenBank under the accession number JX402081. To construct the plant *Nbrgs-CaM* expression vector for transgenic over-expression or transient agroinfiltration assays, a primer pair CaM-F/CaM-R was designed to amplify the full-length coding region of *Nbrgs-CaM*. The *Nbrgs-CaM* coding sequence was subcloned into the binary vector pCHF3 downstream of a CaMV 35S promoter to produce pCHF3-35S-CaM, or into the PVX-based vector (a kind gift of David Baulcombe) between the *Cla*I and *Sal*I sites to produce PVX-Nbrgs-CaM.

An RNAi construct containing an *Nbrgs-CaM* inverted repeat sequence of spaced by a soybean intron was produced by overlapping PCR. The fragment of *Nbrgs-CaM* sense sequence was amplified with A-CaM-F/A-CaM-intron-R primer pair and overlapped with intron sequence amplified by B-CaM-intron-F/B-Intron-R primers. The overlapping products were cloned into pCHF3 between the *Sac*I and *Bam*HI sites to produce pCHF3-35S-CaM-intron. The corresponding antisense Nbrgs-CaM fragment was amplified with the primer pair C-CaM-F/C-CaM-R and subsequently cloned into pCHF3-35S-sCaM-intron between *Bam*HI and *Pst*I sites to produce the RNAi construct pCHF3-35S-dsCaM. To construct a TRV-based recombinant VIGS vector of *Nbrgs-CaM*, a partial fragment of *Nbrgs-CaM* was generated by PCR amplification with the primer pair TRV-CaM-F/TRV-CaM-R and then cloned into the pTRV2 vector (a kind gift of Yule Liu) between the *Bam*HI and *Xho*I sites [54].

The coding sequences of *Ntrgs-CaM* and *Slrgs-CaM* were PCR-amplified from *N. tabacum* and *S. lycopersicum* and then subcloned to the pCHF3 binary vector or the TRV vectors using different primers and restriction enzyme sites listed in Table S1. The pCHF3-based vectors were used for the transient expression of *rgs-CaMs* in *N. benthamiana* leaves, and the TRV-based VIGS vectors were used to silence the expression of *rgs-CaMs* in *N. tabacum* and *S. lycopersicum*, respectively.

Binary vectors for the PTGS suppression assay, such as pCHF3-35S-GFP and pCHF3-35S-dsFP, were constructed previously [82]. The pCHF3-35S-FP vector for transcription of a partial sense sequence of C-terminal fragment of 400 bps of GFP was constructed by amplification with the primer pair S-FP-F/S-FP-R and cloned into pCHF3.

### Plant transformation

Transgenic lines over-expressing or down-regulating *Nbrgs-CaM* were generated by transforming *N. benthamiana* with the pCHF3-CaM and pCHF3-dsCaM constructs, respectively. The binary vectors were mobilized into *A. tumefaciens* EHA105 strain, and then used to transformed *N. benthamiana* leaf discs. Selection of transformants was performed in media containing 200 µg/ml kanamycin. Kanamycin-resistant shootlets were collected, placed on rooting media, grown to a height of 5–6 cm, and then transferred to soil. Transgenic plants were first screened by PCR with specific primers targeted to promoter or intron sequences and then confirmed by Southern blot hybridization. Alterations in *Nbrgs-CaM* mRNA levels in transgenic plants were confirmed by RT-qPCR and Northern blot analyses.

### Viral inoculation and agroinfiltration

For virus agroinoculation, equal volumes of individual *A. tumefaciens* cultures at an OD_600_ of 1 were mixed prior to inoculations. Viral infectious clones, including TYLCCNV (pBinPLUS-Y10-1.7A), and TYLCCNV/TYLCCNB (pBinPLUS-Y10-1.7A+Y10β), were described previously [38]. The TYLCCNV-derived VIGS vectors 2mDNA1 (pBinPLUS-2mDNA1) and 2mDNA1-*NbSu* (pBinPLUS-2mDNA1-*NbSu*) were constructed previously [52], [53]. The recombinant PVX vector expressing *βC1* gene (PVX-βC1) was described previously [66]. *Agrobacterium* cultures carrying viral infectious clone(s) were infiltrated into *N. benthamiana* leaves and inoculated plants were photographed with a Canon 400D digital camera at different periods.

For CMV rub-inoculation, 1 g of CMV-infected *N. glutinosa* leaves were ground in 1 mL of 5 mM phosphate buffer, pH 7.2. *N. benthamiana* plants at the 6–7 leaf stages were inoculated by rubbing leaves with freshly prepared sap. Inoculated plants were grown in an insect-free growth chamber at 25°C and monitored for symptom appearance.

For PTGS experiments, transient silencing suppression assays were performed as described previously [50], [51]. Classic two-component transient PTGS assays were performed by agroinfiltration of 35S:GFP with control or suppressor vectors into leaves of *N. benthamiana* 16c plants at the 6–7 leaf stages. For S-PTGS experiments, *Agrobacterium* cultures harboring the pCHF3-35S-GFP, pCHF3-35S-FP and VSRs-expressing vectors were mixed in equal proportions and infiltrated into *N. benthamiana* leaves. For IR-PTGS experiments, the pCHF3-35S-dsFP was used as an RNA silencing trigger instead of the pCHF3-35S-GF as a RNA silencing trigger. After 3 dpi, GFP fluorescence was monitored with a 100-W handheld long-wavelength UV lamp (Black Ray Model B 100A; UV products) and the infiltrated leaves were photographed with a Canon 400D digital camera with a 58-mm yellow filter. Exposures were 3 to 6 seconds long, depending on the fluorescence intensity and distance from the leaf.

### DNA extraction and Southern blot analysis

Total DNA was extracted from infected plant leaves using the CTAB method (35). DNA agarose gels were stained with ethidium bromide to provide a loading control. After denaturation and neutralization, total DNA was transferred to Hybond N^+^ nylon membranes (GE Healthcare, Pittsburgh, PA) by capillary transfer. Membranes were hybridized at 55°C to specific probes labeled with [α-^32^P] dCTP.

### RNA extraction, northern blot, siRNA blot and RT-qPCR analysis

Total RNAs were extracted from plants with Trizol reagent (Invitrogen, Carlsbad, CA) as recommended by the manufacturer. Total RNA was stained with ethidium bromide as a loading control, and then transferred to Hybond N^+^ nylon membranes by upward capillary transfer in 20×SSC buffer. Membranes were hybridized to specific probes for *rgs-CaM*, *βC1*, *GFP* or PVX *CP* labeled with [α-^32^P] dCTP using random primed labeling System (Promega, Madison, WI). The hybridization signals were detected by phosphorimaging with a Typhoon 9200 imager (GE Healthcare).

To analyze the production siRNAs, low-molecular-mass RNAs were enriched from total RNA as described previously [3]. The enriched small RNAs (15 µg) were fractionated on a 15% denaturing polyacrylamide–7 M urea gel in 0.5× Tris–borate–EDTA (TBE) buffer. The RNA was transferred to Hybond N^+^ membranes (GE Healthcare) by electroblotting in 0.5× TBE at 400 mA for 1 h. The transferred RNAs was UV crosslinked to the membrane 4 times at 1200 µJ in a UV Stratalinker 1800 (Stratagene, La Jolla, CA). Membranes were stored at 4°C until probing. One DNA oligonucleotides complementary to *N. benthamiana U6* RNA and a mixture of oligonucleotides corresponding to G, F and P regions of *GFP* mRNA sequences were synthesized and used as probes for siRNA hybridization (Table S1). The oligos were end-labelled with [γ-^32^P] ATP in 50 µL reactions containing 1 µM DNA oligo and 7 U T4 polynucleotide kinase. Hybridizations were performed overnight at 42°C and the membranes were subsequently washed three times (10 min each) at 40°C with 1× SSC (0.15 M NaCl and 0.015 M sodium citrate) supplemented with 0.1% SDS. Hybridization signals were detected as described above for Northern blot analysis.

For RT-qPCR analysis, 10 µg of total RNA was treated with DNase I (Takara) and reverse transcribed according to manufacturer's instructions. Specific primer pairs, which annealed to *GFP*, *βC1*, *Nbrgs-CaM*, *Su* or genes encoding known components in RNA silencing pathway (Table S1), were designed by Primer Premier 5 software. The GenBank accession numbers of genes analyzed in this study are as follows: *Ntrgs-CaM* (AF329729), *Slrgs-CaM* (AY642285), *NbDCL1* (FM986780), *NbDCL2* (FM986781), *NbDCL3* (FM986782), *NbDCL4* (FM986783), *NbAGO1-1* (DQ321488), *NbAGO4-1* (DQ321490), *NtSGS3a* (AB690269), *NbRDR1m* (AY574374), *NbRDR2* (AY722009), *NbRDR6* (AY722008). The lengths of amplification products were between 180–250 bp, and the Tm for each primer pair was between 55–65°C. RT-qPCR was performed using a LightCycler 480 (Roche Diagnostics, Rotkreuz, Switzerland) for 45 cycles, and *NbGAPDH* was used an internal control unless otherwise stated. Each experiment was performed in triplicate and repeated three times, and the results were analyzed by software supplied by the manufacturer.

### Immunoblotting and antibodies

Total proteins were extracted from *N. benthamiana* leaves as described previously [66]. Immunoblotting was performed with primary mouse monoclonal or rabbit polyclonal antibodies, followed by goat anti-mouse or anti-rabbit secondary antibody conjugated to horseradish peroxidase (Bio-Rad, Hercules, CA). The GFP monoclonal antibody was obtained from Hua An Company, China, and the monoclonal antibodies against PVX CP, CMV CP and βC1 were generated in house. The rabbit polyclonal antibody against CMV 2b was a generous gift from Dr. Huishan Guo. Blotted membranes were washed thoroughly and visualized using chemiluminescence according to the manufacturer's manual (GE Healthcare). Nbrgs-CaM antibody was produced in rabbit with recombinant protein expressed in *E. coli*. In brief, we expressed *Nbrgs-CaM* in E. coli cells as a 6× Histidine and Maltose binding protein (MBP) fusion. The Nbrgs-CaM-MBP-His fusion protein was purified over a nickle column (Merck, Darmstadt, Germany) and eluted with a buffer containing 200 mM imidazole according to the manufacturer's manual. The purified protein was used to immunize rabbits for polyclonal antiserum. The Nbrgs-CaM-specific immunoglubin was given an additional purification step on an affinity column filled with Cyanogen bromide-activated agarose beads conjugated with the Nbrgs-CaM-MBP-His fusion proteins.

## Supporting Information

Figure S1 **Induction of** ***rgs-CaM*** **s by TYLCCNV betasatellite in** ***Nicotiana benthamiana, N. tabacum*** **, and** ***Solanum lycopersicum*** **.** Levels of *Nbrgs-CaM* (left), *Ntrgs-CaM* (middle) and *Slrgs-CaM* (right) mRNAs in mock, 10- and 10Aβ-infected plants at 12 dpi. Mock indicates plants infiltrated with *agrobacterium* carrying an empty vector. The levels of *rgs-CaM* mRNA were separately normalized to *NbGAPDH, NtEF-1-α* or S*l EF-1-α* that served as an internal control. The values in the mock plants were arbitrarily set to 1. Error bars represent SD of nine biological replicates and asterisk indicates P value between treatments: *P≤0.05 (Student's *t* test).
(TIF)

Figure S2 **Nbrgs-CaM negatively regulates VIGS of an endogenous gene in** ***Nicotiana benthamiana*** **.** (**A**) Phenotypes of *Su* VIGS. Wt *N. benthamiana, Nbrgs-CaM*-overexpression lines (35S:CaM) and RNAi line (35S:dsCaM) plants infected with the TYLCCNV-derived VIGS vector carrying a portion of the *Su* gene (10A+2mDNA1-*NbSu*) and photographed at 30 dpi. VIGS of *Su* results in yellow-white spots phenotype in wt plants. *Su*-silencing phenotypes were enhanced in 35S:dsCaM-#2 plants but inhibited in 35S:CaM lines #1.3, #1.5, #2.4 and #2.6. Mock represents wt plants infected by the VIGS vector without *Su* insertion (10A+2mDNA1). (**B**) Levels of *Su* mRNA in inoculated and systemically infected leaves. The levels of *Su* mRNA were analyzed by RT-qPCR and normalized to mRNA of *NbGAPDH* that served as an internal standard. The value in mock plants was arbitrarily designed as 1. Error bars: ± SD. Asterisks indicate P value compared with mock-treated wild type plants: *P≤0.05, **P≤0.01 (Student's *t* test).
(TIF)

Figure S3 **Ntrgs-CaM and Slrgs-CaM suppress S-PTGS but not IR-PTGS.** (**A**) and (**B**) GFP fluorescence in leaves of *Nicotiana benthamiana* plants co-infiltrated *Agrobacterium* cultures expressing the indicated suppressors and GFP reporter (35S:GFP), together with either a sense RNA fragment of GFP (35S:FP) (**A**) or dsRNA fragment of GFP (35S:dsFP) (**B**) as indicated on top of each panel. The infiltrated leaves were photographed under UV light at 3 and 5 dpi. (**C**) and (**D**) Accumulation of GFP and βC1 protein, GFP and rgs-CaM mRNA, GFP siRNA and U6 RNA in agroinfiltrated leaves shown in (**A**) and (**B**), respectively. GFP or βC1 specific monoclonal antibody was used in immunoblotting. Coomassie blue staining of the large subunit of Rubisco served as loading controls. In large RNA blot, [α-^32^P]-labeled DNA fragments of *GFP* and *Nbrgs-CaM* were used as probes and ethidium bromide staining of 25S rRNA indicated the equal loading. For the small RNA blot, [γ-^32^P] ATP-labeled GFP DNA oligonucleotides annealed to different region of GFP mRNA were mixed and used as probes. U6 RNA hybridizations were used as a loading control of the small RNA blot. The sizes of the 21-, 22- and 24-nt RNAs are indicated to the right of the small RNA panel.
(TIF)

Figure S4 **Knockdown of** ***Nbrgs-CaM*** **expression in** ***Nicotiana benthamiana*** **abolishes βC1 VSR activity.** The *N. benthamiana* 16c plants were inoculated at 4–5 leaf stage with a recombinant *Tobacco rattle virus* (TRV) vector harboring a partial sequence of *Nbrgs-CaM* (TRV-CaM). At 7 dpi, the *Nbrgs-CaM* mRNAs in upper emerged leaves were assessed by RT-qPCR. The leaves of *Nbrgs-CaM*-silenced plants or mock-silenced plants (TRV-inoculated) were assayed for PTGS suppression. (**A**) GFP fluorescence in leaves of mock-silenced and *Nbrgs-CaM*-silenced plants co-infiltrated with 35S:GFP and indicated suppressors or vector control. Photographs were taken under UV light at 3 dpi (left panels) and 5 dpi (right panels). (**B**) RT-qPCR analysis of *Nbrgs-CaM* mRNA in systemically infected leaves of plants inoculated by TRV and TRV-CaM at 7 dpi. Error bars: ± SD. Asterisks indicate P value compared with mock-treated wild type plants: *P≤0.05, **P≤0.01 (Student's *t* test). (**C**) Accumulations of GFP and βC1 protein, GFP mRNA and siRNA in infiltrated leaves shown in (**A**) at 5 dpi. Protein levels were analyzed in immunoblots using GFP or βC1 specific monoclonal antibody. Coomassie blue staining of the large subunit of Rubisco served as loading controls. In large RNA blot, [α-^32^P]-labeled DNA fragments of *GFP* and *Nbrgs-CaM* were used as probes and ethidium bromide staining of 25S rRNA were used to show the equal loading. In the small RNA blot, [γ-^32^P]-labeled GFP or U6 oligonucleotides were used as probes in small RNA blot. The sizes of the 21-, 22- and 24-nt RNAs are indicated.
(TIF)

Figure S5 **Nbrgs-CaM positively regulates the symptoms and accumulation of CMV.** (**A**) Symptoms of *Nicotiana benthamiana* wt, *Nbrgs-CaM*-overexpression (35S:CaM) and RNAi (35S:dsCaM) plants infected by CMV at 12 (upper row) and 20 dpi (bottom row). (**B**) Western blots of CMV CP, 2b and actin protein accumulation in systemically infected leaves at 12 and 20 dpi. Coomassie blue (CB) staining of total protein gels serve as loading controls.
(TIF)

Figure S6 **Efficient reduction of** ***rgs-CaM*** **expression by TRV-VIGS vector in** ***Nicotiana benthamiana, N. tabacum and Solanum lycopersicum*** **.** Partial fragments of *Nbrgs-CaM*, *Ntrgs-CaM* and *Slrgs-CaM* were cloned into the RNA2 of TRV VIGS vector (pTRV2). The pTRV2 with a GFP insertion (pTRV-GFP) was used as a negative control. *N. benthamiana* (*Nb*), *N. tabacum* (*Nt*), *S. lycopersicum* (*Sl*) plants at 4–5 leaf stage were infiltrated with *Agrobacterium* cultures carrying pTRV1 with either an empty pTRV2 (Mock), with pTRV2-GFP, or with the respective pTRV2-CaM vectors. The mRNA levels of *Nbrgs-CaM*, *Ntrgs-CaM* and *Slrgs-CaM* were analyzed by RT-qPCR using specific primers and then normalized to *NbGAPDH* mRNA for *N. benthamiana*, or to *EF-1-α* mRNA for *N. tabacum* and *S. lycopersicum*. Error bars represent SD of nine biological replicates and asterisks indicate P values between treatments: *P≤0.05, **P≤0.01 (Student's *t* test).
(TIF)

Figure S7 **Similar effects of 35S:CaM and dsRDR6 plants on suppression of S-PTGS, but not IR-PTGS.** (**A**) GFP fluorescence of leaves of wt, 35S:CaM and dsRDR6 *Nicotiana benthamiana* plants co-infiltrated with *Agrobacterium* cultures expressing the GFP reporter (35S:GFP) together with either the sense-PTGS trigger (35S:FP) or inverted repeat of GFP fragment (35S:dsFP) as indicated on the top of each panel. Wt plants were infiltrated with bacterium cultures expressing the GFP reporter and silencing triggers, vector control (Vec) or *p19* as indicated. Photographs were taken 5 dpi under UV light. (**B**) Accumulation of GFP in infiltrated leaves using a GFP-specific antibody in Western blots (WB). Coomassie blue staining of the large subunit of Rubisco served as a loading control.
(TIF)

Table S1 **Primers used in plasmid construction and other experiments.**
(DOCX)
