# Supplementary figures and images for: Suppression of RNA Silencing by a Plant DNA Virus Satellite Requires a Host Calmodulin-Like Protein to Repress RDR6 Expression
Source: PLoS Pathog. 2014 Feb 6;10(2):e1003921. doi: 10.1371/journal.ppat.1003921 (PMC3916407; doi:10.1371/journal.ppat.1003921)

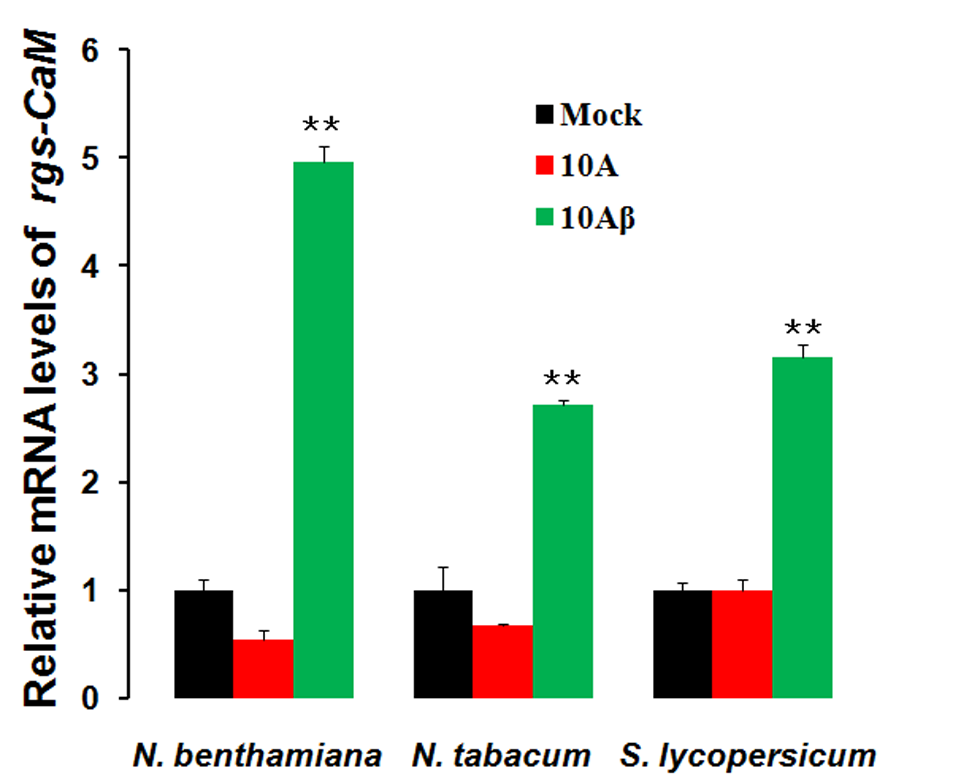

Supplement: Figure S1 — Induction of rgs-CaM s by TYLCCNV betasatellite in Nicotiana benthamiana, N. tabacum , and Solanum lycopersicum . Levels of Nbrgs-CaM (left), Ntrgs-CaM (middle) and Slrgs-CaM (right) mRNAs in mock, 10- and 10Aβ-infected plants at 12 dpi. Mock indicates plants infiltrated with agrobacterium carrying an empty vector. The levels of rgs-CaM mRNA were separately normalized to NbGAPDH, NtEF-1-α or Sl EF-1-α that served as an internal control. The values in the mock plants were arbitrarily set to 1. Error bars represent SD of nine biological replicates and asterisk indicates P value between treatments: *P≤0.05 (Student's t test). (TIF) [file ppat.1003921.s001.tif]

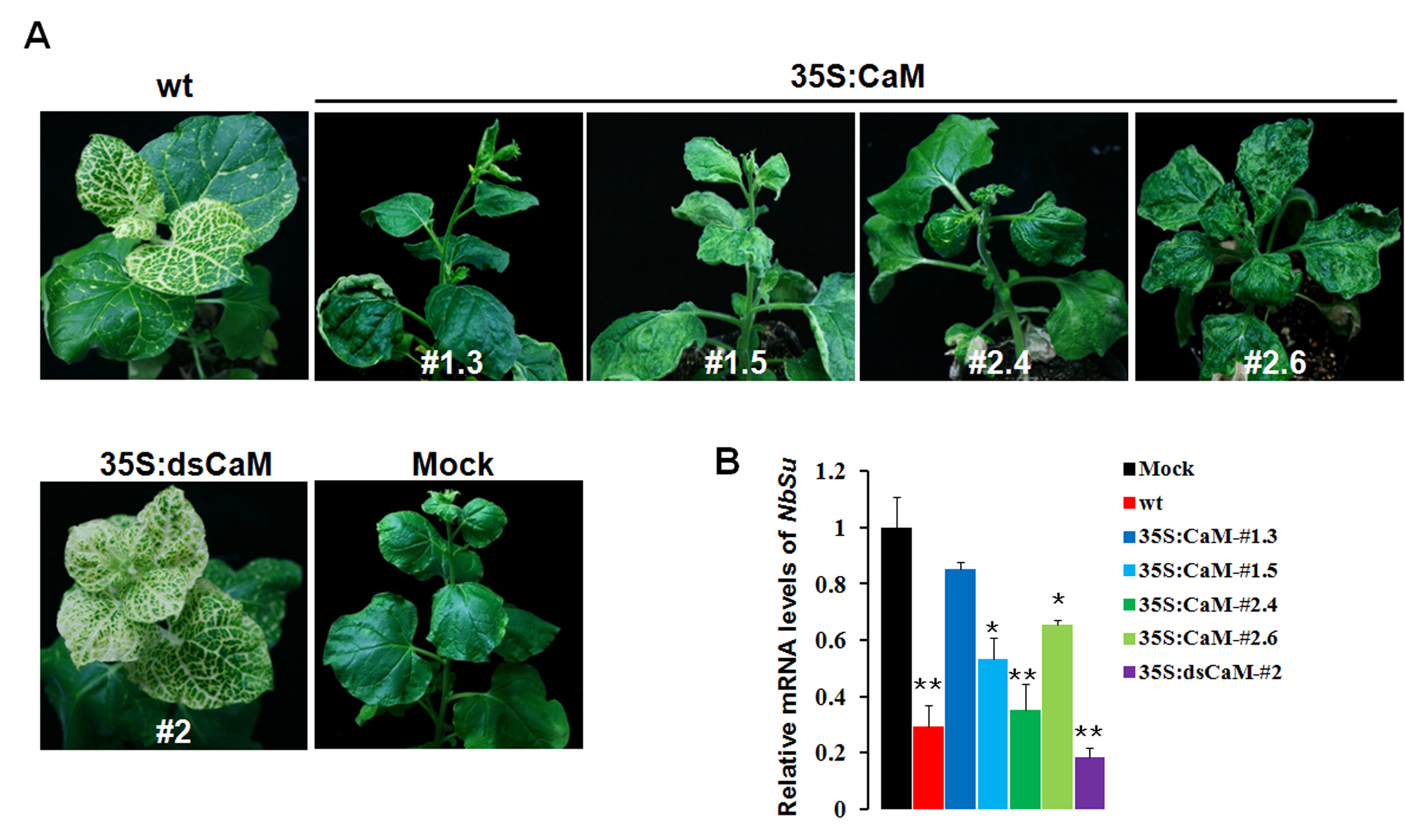

Supplement: Figure S2 — Nbrgs-CaM negatively regulates VIGS of an endogenous gene in Nicotiana benthamiana . (A) Phenotypes of Su VIGS. Wt N. benthamiana, Nbrgs-CaM-overexpression lines (35S:CaM) and RNAi line (35S:dsCaM) plants infected with the TYLCCNV-derived VIGS vector carrying a portion of the Su gene (10A+2mDNA1-NbSu) and photographed at 30 dpi. VIGS of Su results in yellow-white spots phenotype in wt plants. Su-silencing phenotypes were enhanced in 35S:dsCaM-#2 plants but inhibited in 35S:CaM lines #1.3, #1.5, #2.4 and #2.6. Mock represents wt plants infected by the VIGS vector without Su insertion (10A+2mDNA1). (B) Levels of Su mRNA in inoculated and systemically infected leaves. The levels of Su mRNA were analyzed by RT-qPCR and normalized to mRNA of NbGAPDH that served as an internal standard. The value in mock plants was arbitrarily designed as 1. Error bars: ± SD. Asterisks indicate P value compared with mock-treated wild type plants: *P≤0.05, **P≤0.01 (Student's t test). (TIF) [file ppat.1003921.s002.tif]

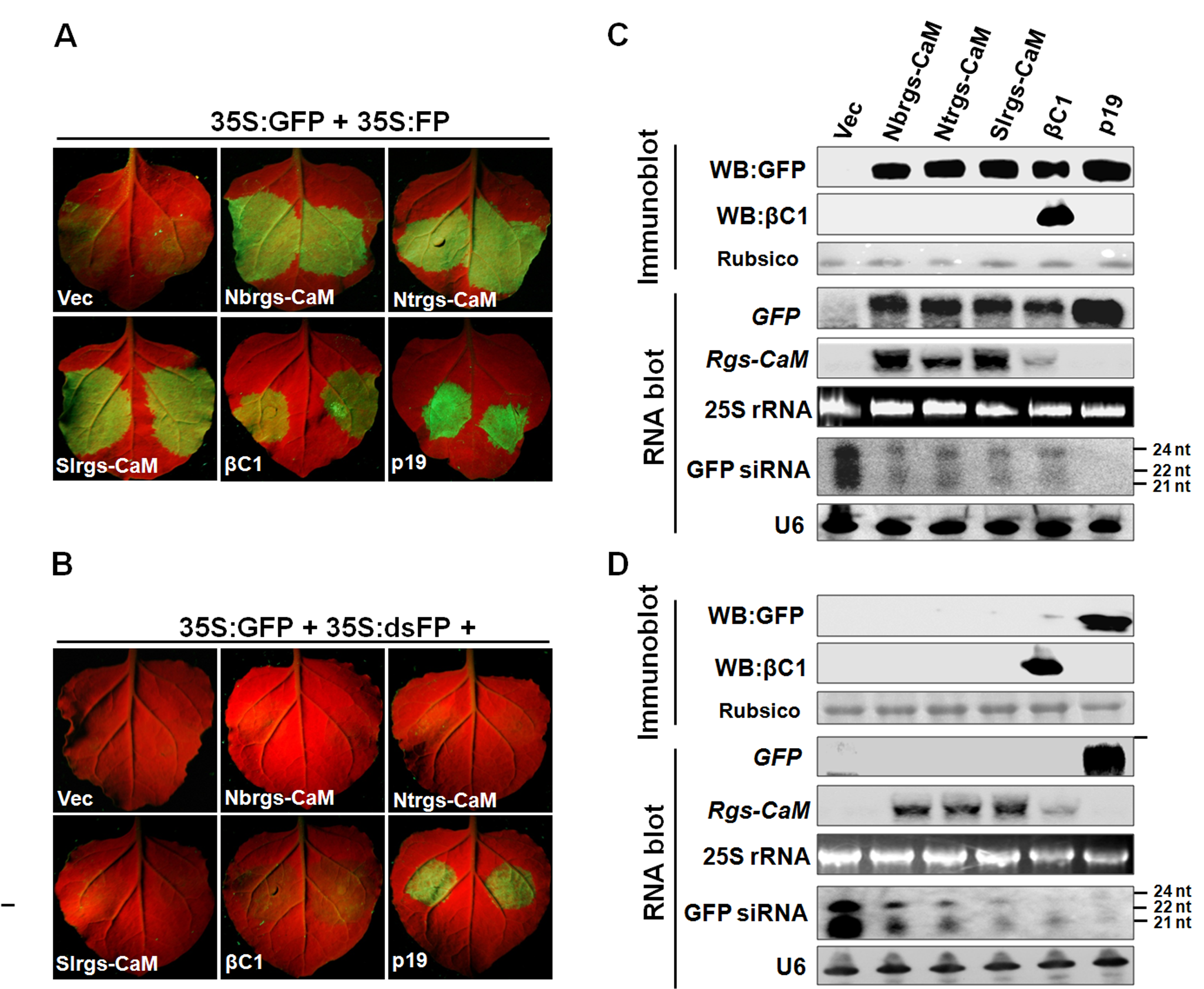

Supplement: Figure S3 — Ntrgs-CaM and Slrgs-CaM suppress S-PTGS but not IR-PTGS. (A) and (B) GFP fluorescence in leaves of Nicotiana benthamiana plants co-infiltrated Agrobacterium cultures expressing the indicated suppressors and GFP reporter (35S:GFP), together with either a sense RNA fragment of GFP (35S:FP) (A) or dsRNA fragment of GFP (35S:dsFP) (B) as indicated on top of each panel. The infiltrated leaves were photographed under UV light at 3 and 5 dpi. (C) and (D) Accumulation of GFP and βC1 protein, GFP and rgs-CaM mRNA, GFP siRNA and U6 RNA in agroinfiltrated leaves shown in (A) and (B), respectively. GFP or βC1 specific monoclonal antibody was used in immunoblotting. Coomassie blue staining of the large subunit of Rubisco served as loading controls. In large RNA blot, [α-32P]-labeled DNA fragments of GFP and Nbrgs-CaM were used as probes and ethidium bromide staining of 25S rRNA indicated the equal loading. For the small RNA blot, [γ-32P] ATP-labeled GFP DNA oligonucleotides annealed to different region of GFP mRNA were mixed and used as probes. U6 RNA hybridizations were used as a loading control of the small RNA blot. The sizes of the 21-, 22- and 24-nt RNAs are indicated to the right of the small RNA panel. (TIF) [file ppat.1003921.s003.tif]

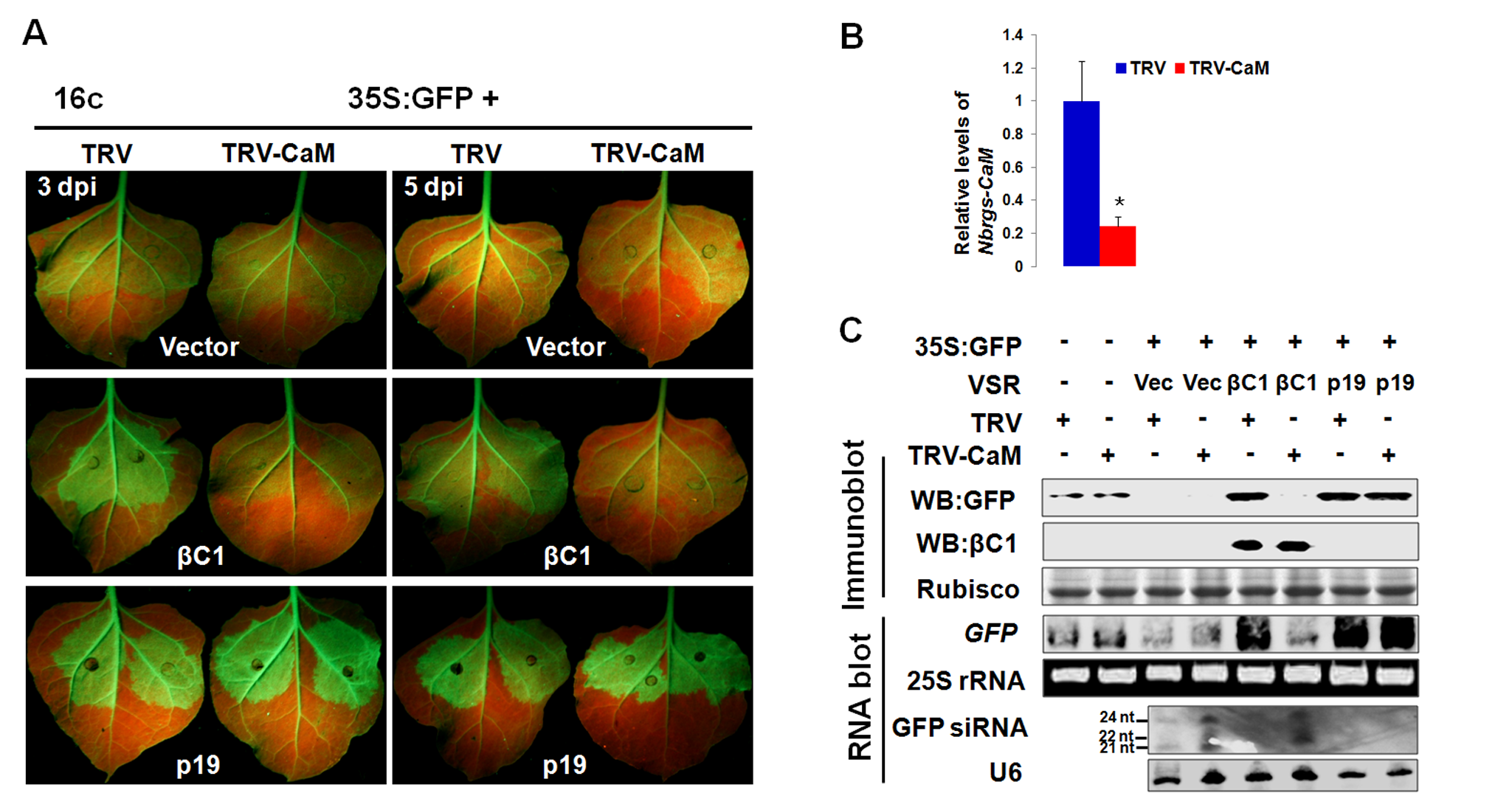

Supplement: Figure S4 — Knockdown of Nbrgs-CaM expression in Nicotiana benthamiana abolishes βC1 VSR activity. The N. benthamiana 16c plants were inoculated at 4–5 leaf stage with a recombinant Tobacco rattle virus (TRV) vector harboring a partial sequence of Nbrgs-CaM (TRV-CaM). At 7 dpi, the Nbrgs-CaM mRNAs in upper emerged leaves were assessed by RT-qPCR. The leaves of Nbrgs-CaM-silenced plants or mock-silenced plants (TRV-inoculated) were assayed for PTGS suppression. (A) GFP fluorescence in leaves of mock-silenced and Nbrgs-CaM-silenced plants co-infiltrated with 35S:GFP and indicated suppressors or vector control. Photographs were taken under UV light at 3 dpi (left panels) and 5 dpi (right panels). (B) RT-qPCR analysis of Nbrgs-CaM mRNA in systemically infected leaves of plants inoculated by TRV and TRV-CaM at 7 dpi. Error bars: ± SD. Asterisks indicate P value compared with mock-treated wild type plants: *P≤0.05, **P≤0.01 (Student's t test). (C) Accumulations of GFP and βC1 protein, GFP mRNA and siRNA in infiltrated leaves shown in (A) at 5 dpi. Protein levels were analyzed in immunoblots using GFP or βC1 specific monoclonal antibody. Coomassie blue staining of the large subunit of Rubisco served as loading controls. In large RNA blot, [α-32P]-labeled DNA fragments of GFP and Nbrgs-CaM were used as probes and ethidium bromide staining of 25S rRNA were used to show the equal loading. In the small RNA blot, [γ-32P]-labeled GFP or U6 oligonucleotides were used as probes in small RNA blot. The sizes of the 21-, 22- and 24-nt RNAs are indicated. (TIF) [file ppat.1003921.s004.tif]

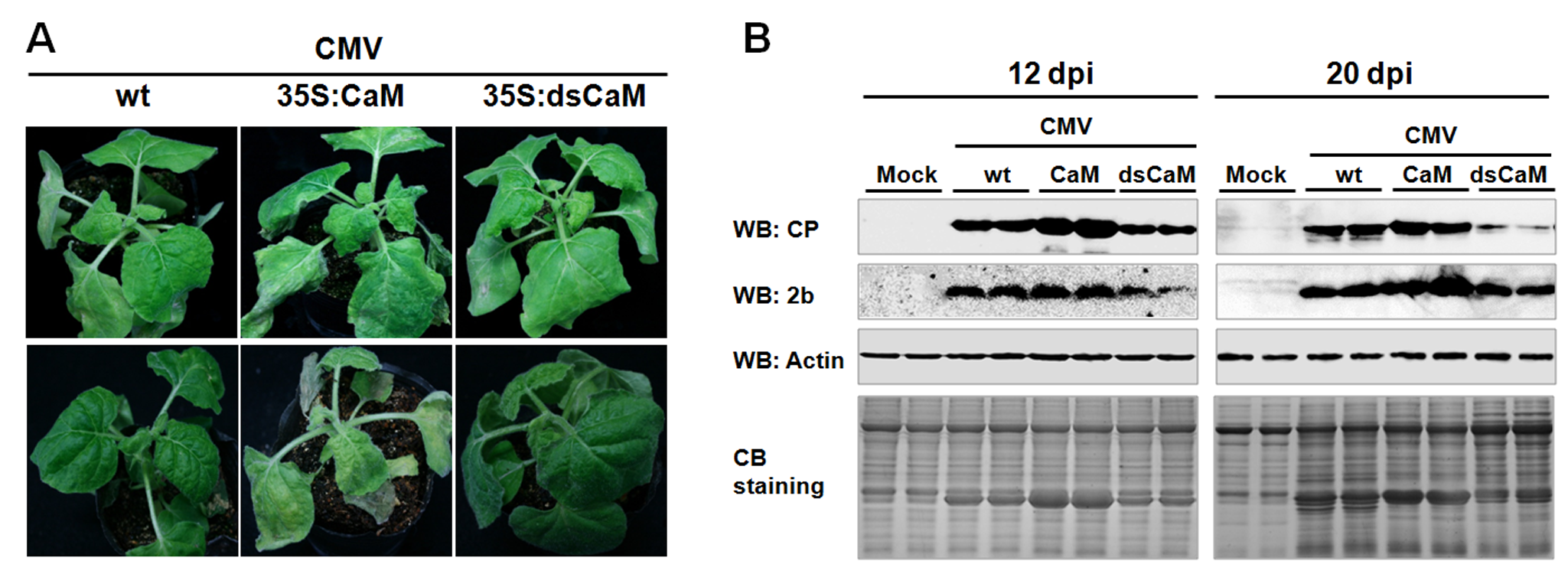

Supplement: Figure S5 — Nbrgs-CaM positively regulates the symptoms and accumulation of CMV. (A) Symptoms of Nicotiana benthamiana wt, Nbrgs-CaM-overexpression (35S:CaM) and RNAi (35S:dsCaM) plants infected by CMV at 12 (upper row) and 20 dpi (bottom row). (B) Western blots of CMV CP, 2b and actin protein accumulation in systemically infected leaves at 12 and 20 dpi. Coomassie blue (CB) staining of total protein gels serve as loading controls. (TIF) [file ppat.1003921.s005.tif]

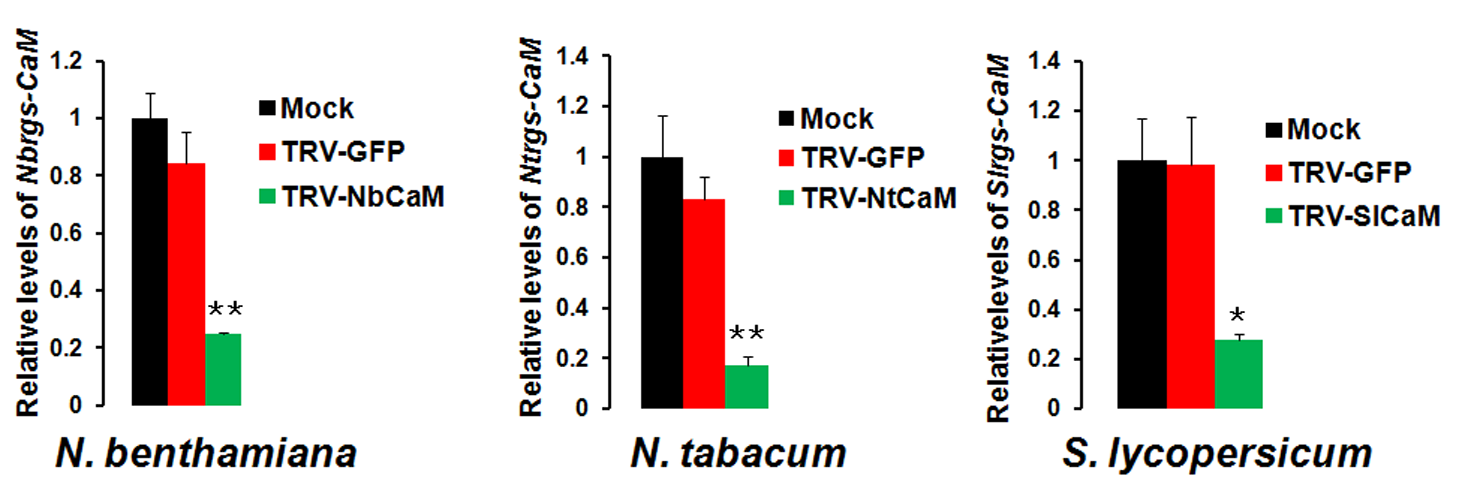

Supplement: Figure S6 — Efficient reduction of rgs-CaM expression by TRV-VIGS vector in Nicotiana benthamiana, N. tabacum and Solanum lycopersicum . Partial fragments of Nbrgs-CaM, Ntrgs-CaM and Slrgs-CaM were cloned into the RNA2 of TRV VIGS vector (pTRV2). The pTRV2 with a GFP insertion (pTRV-GFP) was used as a negative control. N. benthamiana (Nb), N. tabacum (Nt), S. lycopersicum (Sl) plants at 4–5 leaf stage were infiltrated with Agrobacterium cultures carrying pTRV1 with either an empty pTRV2 (Mock), with pTRV2-GFP, or with the respective pTRV2-CaM vectors. The mRNA levels of Nbrgs-CaM, Ntrgs-CaM and Slrgs-CaM were analyzed by RT-qPCR using specific primers and then normalized to NbGAPDH mRNA for N. benthamiana, or to EF-1-α mRNA for N. tabacum and S. lycopersicum. Error bars represent SD of nine biological replicates and asterisks indicate P values between treatments: *P≤0.05, **P≤0.01 (Student's t test). (TIF) [file ppat.1003921.s006.tif]

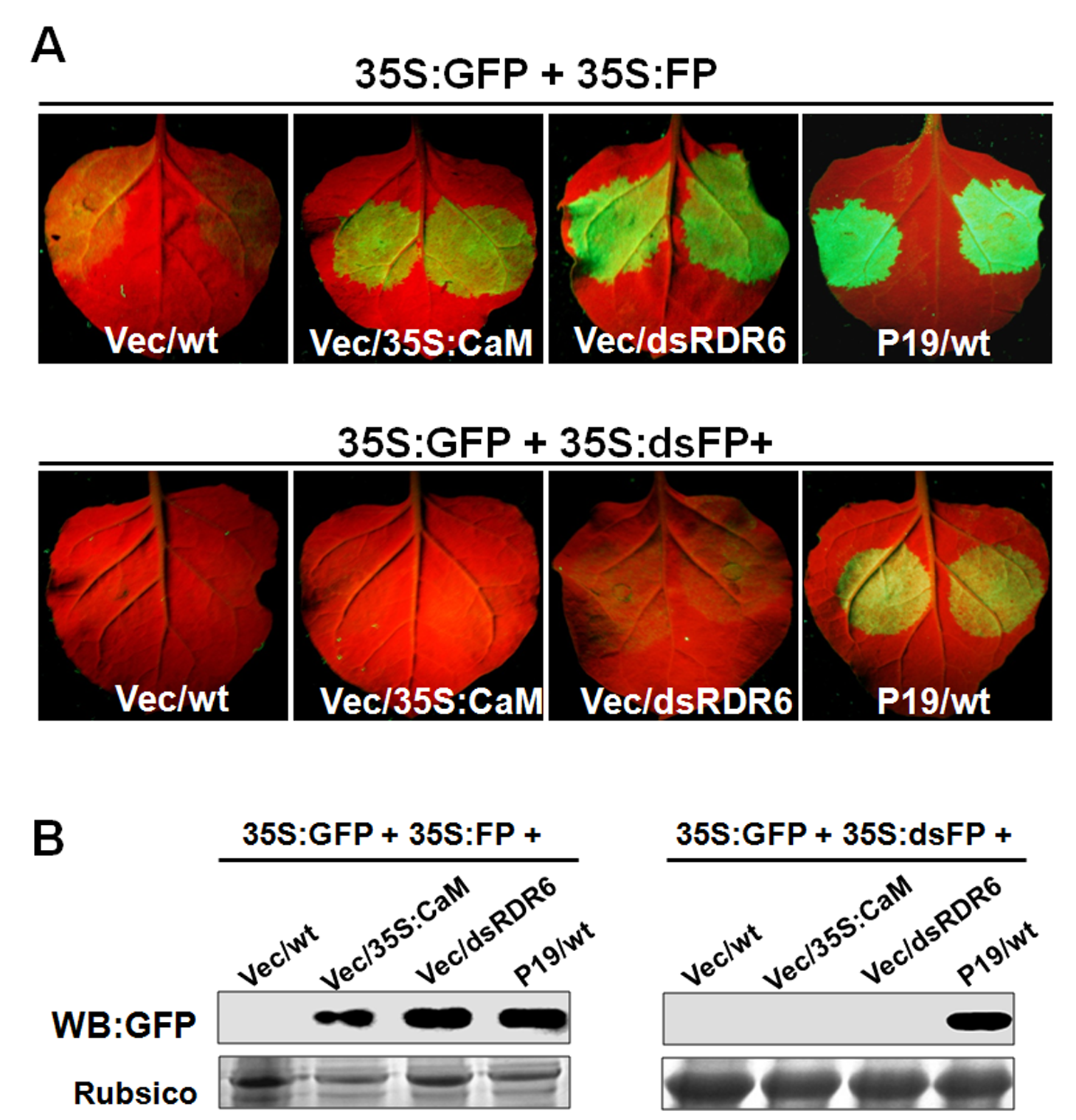

Supplement: Figure S7 — Similar effects of 35S:CaM and dsRDR6 plants on suppression of S-PTGS, but not IR-PTGS. (A) GFP fluorescence of leaves of wt, 35S:CaM and dsRDR6 Nicotiana benthamiana plants co-infiltrated with Agrobacterium cultures expressing the GFP reporter (35S:GFP) together with either the sense-PTGS trigger (35S:FP) or inverted repeat of GFP fragment (35S:dsFP) as indicated on the top of each panel. Wt plants were infiltrated with bacterium cultures expressing the GFP reporter and silencing triggers, vector control (Vec) or p19 as indicated. Photographs were taken 5 dpi under UV light. (B) Accumulation of GFP in infiltrated leaves using a GFP-specific antibody in Western blots (WB). Coomassie blue staining of the large subunit of Rubisco served as a loading control. (TIF) [file ppat.1003921.s007.tif]
