# Supplementary material for: Suppression of RNA Silencing by a Plant DNA Virus Satellite Requires a Host Calmodulin-Like Protein to Repress RDR6 Expression
Source: PLoS Pathog. 2014 Feb 6;10(2):e1003921. doi: 10.1371/journal.ppat.1003921 (PMC3916407; doi:10.1371/journal.ppat.1003921)
Supplement: Table S1 — Primers used in plasmid construction and other experiments. (DOCX) [file ppat.1003921.s008.docx]

Table S1. Primers used in plasmid construction and other experiments.

| Primer | Sequence | Purpose |
| --- | --- | --- |
| NbCaM-cds-F | 5'-TTACTAGTTTAGTTGTTATACG-3' | To amplify the complete coding region of Nbrgs-CaM |
| NbCaM-cds-R | 5'-CCAAGGCCAAAGAATTATGTACATG-3' | To amplify the complete coding region of Nbrgs-CaM |
| NbCaM-F | 5'-GGATCCATGTGCATGGAATCA-3' | pCHF3-35S-NbCaM, for transgenic over-expression and transient agro-infiltration |
| NbCaM-R | 5'-CTGCAGTTAACTTGTCATCATAG-3' | pCHF3-35S-NbCaM, for transgenic over-expression and transient agro-infiltration |
| NtCaM-F | 5'-CGGGGTACCATGGAATCAGTTTCTGTACC-3' | pCHF3-35S-NtCaM, for transient agro-infiltration |
| NtCaM-R | 5'-AACTGCAGTTAACTTGTCATCATAGCTTTG-3' | pCHF3-35S-NtCaM, for transient agro-infiltration |
| SlCaM-F | 5'-CGGGGTACCATGTCTAATATGGTGTTAGTTAC-3' | pCHF3-35S-SlCaM, for transient agro-infiltration |
| SlCaM-R | 5'-AACTGCAGCTAACTTGTCATCATAACTTTG-3' | pCHF3-35S-SlCaM, for transient agro-infiltration |
| TRV-NbCaM-F | 5'-CGGAATTCTCTAGAAATCAGTTTCTGTACCTAGTGTTG3' | pTRV-NbCaM, for VIGS of Nbrgs-CaM via TRV vector |
| TRV-NbCaM-R | 5'-CCGCTCGAGCATCGCCGTCGGAATCCGATAGTC-3' | pTRV-NbCaM, for VIGS of Nbrgs-CaM via TRV vector |
| TRV-NtCaM-F | 5'-CGGAATTCTCTAGATTTCTGTACCTAGTGTTGAAAAC-3' | pTRV-NtCaM, for VIGS of Nbrgs-CaM via TRV vector |
| TRV-NtCaM-R | 5'-CCGCTCGAGCATCCCCGTCGGAATCCGATAGCC-3' | pTRV-NtCaM, for VIGS of Nbrgs-CaM via TRV vector |
| TRV-SlCaM-F  TRV-SlCaM-R | 5'-CGGAATTCTCTAGATGGTGTTAGTTACTGTTTCCATGG-3'  5'-CCGCTCGAGCCATCTCCGCCTCCTCCACCG-3' | pTRV-SlCaM, for VIGS of Nbrgs-CaM via TRV vector  pTRV-SlCaM, for VIGS of Nbrgs-CaM via TRV vector |
| A-CaM-F | 5'-GAGCTCTGTGCATGGAATCAGTTTCTGTACC-3' | pCHF3-35S-dsCaM, for down-regulation of Nbrgs-CaM |
| A-CaM-intron-R | 5'-ATCAGACTTACAACGTATACATTCCAAATGCT-3' | pCHF3-35S-dsCaM, for transgenic down-regulation of Nbrgs-CaM |
| B- CaM-intron-F | 5'-AGCATTTGGAATGTATACGTTGTAAGTCTGAT-3' | pCHF3-35S-dsCaM, for transgenic down-regulation of Nbrgs-CaM |
| B-Intron-R | 5'-GGATCCGCTCTATCTGCTGGGTCCAAATC-3' | pCHF3-35S-dsCaM, for transgenic down-regulation of Nbrgs-CaM |
| C-CaM-F | 5'-GGATCCATACATTCCAAATGCTCCTATC-3' | pCHF3-35S-dsCaM, for transgenic down-regulation of Nbrgs-CaM |
| C-CaM-R | 5'-CTGCAGTGTGCATGGAATCAGTTTCTG-3' | pCHF3-35S-dsCaM, for transgenic down-regulation of Nbrgs-CaM |
| PVX-CaM-F | 5'-ATCGATATGTGCATGGAATCAAGTTTC-3' | pGR-PVX-CaM, for over-expression of Nbrgs-CaM via PVX vector |
| PVX-CaM-R | 5'-GTCGACTTAACTTGTCATCATAGCTTTG-3' | pGR-PVX-CaM, for over-expression of Nbrgs-CaM via PVX vector |
| S-FP-F | 5'-GAGCTCCAGTGGAGAGGGTGAAGGTGATGC-3' | pCHF3-FP, for inducing GFP silencing |
| S-FP-R | 5'-GGATCCCTTTGATTCCATTCTTTTGTTTG-3' | pCHF3-FP, for inducing GFP silencing |
| Y10A-F | 5'-ATGTGGGATCCTCTGCTCAACGAGTTTC-3' | To make probe for the detection of TYLCCNV in Southern blot |
| Y10A-R | 5'-CATCCTCAGACCTTGCGTTTCTTAAGAG-3' | To make probe for the detection of TYLCCNV in Southern blot |
| Y35DNA1-F | 5'-CCATCGATATGCCTTCAATTACCTCTGTC-3' | To make probe for the detection of DNA1 in Southern blot |
| Y35DNA1-R | 5'-GGTGTCGACTTAGTCCAAATACTCGTCGC-3' | To make probe for the detection of DNA1 in Southern blot |
| 35S-F | 5'-ACATGGTGGAGCACGACACG-3' | PCR screening of transgenic plants containing CaMV 35S promoter |
| 35S-R | 5'-GAGGAAGGGTCTTGCGAAGG-3' | PCR screening of transgenic plants containing CaMV 35S promoter |
| CaM-p-F | 5'-GGATCCTAATACGACTCACTATAGGGAGAAATCCTCCAAACCCAACA-3' | To make probe for the detection of Nbrgs-CaM in Northern blot |
| CaM-p-R | 5'-TTCACTCAAAAAGGCAACGACAACAACAACTATTAC-3' | To make probe for the detection of Nbrgs-CaM in Northern blot |
| βC1-p-F | 5'-GGATCCTAATACGACTCACTATAGGGAGAACATCATACTCATCCCCT-3' | To make probe for the detection of βC1 in Northern blot |
| βC1-p-R | 5'-AGTCACCAGCATTAGCCAAGAAGAAATTCATG-3' | To make probe for the detection of βC1 in Northern blot |
| GFP-p-F | 5'-CATGAGTAAAGGAGAAGAACTTTTC-3' | To make probe for the detection of GFP in Northern blot |
| GFP-p-R | 5'-TTCATATGATCTGGGTATCTTG-3' | To make probe for the detection of βC1 in Northern blot |
| PVX-CP-F | 5'-CACAACACAGCCCATAGGGTC-3' | To make probe for the detection of PVX CP in Northern blot |
| PVX-CP-R | 5'-TTAACATCCAGTTCCATACCACTG-3' | To make probe for the detection of PVX CP in Northern blot |
| U6 | 5'-TTGCGTGTCATCCTTGCGCAGG-3 | DNA oligo probe for the detection of U6 in siRNAs blot |
| G1 | 5'-GGATCCAAGGAGATATAACAATGAAGACTAATCTTTTTCT-3 | DNA oligo probe for the detection of G siRNAs |
| G2 | 5'-CTTTCTCATCTTTTCACTTCTCCTATCATTATCCTCGGCC-3 | DNA oligo probe for the detection of G siRNAs |
| G3 | 5'-GAATTCAGTAAAGGAGAAGAACTTTTCACTGGAGTTGTC-3 | DNA oligo probe for the detection of G siRNAs |
| F1 | 5'-AATTTTCTGTCAGTGGAGAGGGTGAAGGTGATGCAACATA-3 | DNA oligo probe for the detection of F siRNAs |
| F2 | 5'-CGGAAAACTTACCCTTAAATTTATTTGCACTACTGGAAAA-3 | DNA oligo probe for the detection of F siRNAs |
| F3 | 5'-CTACCTGTTCCATGGCCAACACTTGTCACTACTTTCTCTT-3 | DNA oligo probe for the detection of F siRNAs |
| P1 | 5'-CAAGTTGGAATACAACTACAACTCCCACAACGTATACATC-3 | DNA oligo probe for the detection of P siRNAs |
| P2 | 5'-ATGGCAGACAAACAAAAGAATGGAATCAAAGTTAACTTCA-3 | DNA oligo probe for the detection of P siRNAs |
| P3 | 5'-AAATTAGACACAACATTGAAGATGGAAGCGTTCAACTAGC-3 | DNA oligo probe for the detection of P siRNAs |
| Y10A-1-siRNA  Y10A-2-siRNA  Y10A-3-siRNA | 5'-CCCCTATCACCCATTATCATCAC-3'  5'-TTGCAGTTAGTTGAAAAAACCTACTCTCCTG-3'  5'-CCCGCAGATATAGTCATTTCCACTCCCGCCT-3' | DNA oligo probe for the detection of TYLCCNV siRNAs  DNA oligo probe for the detection of TYLCCNV siRNAs  DNA oligo probe for the detection of TYLCCNV siRNAs |
| Beta-1-siRNA | 5'-TCATATATTTGAATACGTATATATATGTATT-3' | DNA oligo probe for the detection of TYLCCNB siRNAs |
| Beta-2-siRNA | 5'-GAATTTGTAAATACATCATACTCATCCCCTA-3' | DNA oligo probe for the detection of TYLCCNB siRNAs |
| Beta-3-siRNA | 5'-GGTGGGATAATCCCATGGTGTCTGTATGGGA-3' | DNA oligo probe for the detection of TYLCCNB siRNAs |
| NbSu-1-siRNA | 5'-CAGGGCAGAGTCAAGGGAGGAAGTTTTATGGAGGGATTAG-3' | DNA oligo probe for the detection of NbSu siRNAs |
| NbSu-2-siRNA | 5'-AAGTCAGCCCTGCTCAAGAACAGGGTCAGAAACTTGCTGA-3' | DNA oligo probe for the detection of NbSu siRNAs |
| NbSu-3-siRNA | 5'-ATGTCTTTTGCTGAATGTAATTGATCCAAAGATTGGAGGT-3' | DNA oligo probe for the detection of NbSu siRNAs |
| GAPDH-q-F | 5'-GCAGTGAACGACCCATTTATCTC-3' | Relative qRT-PCR analysis of NbGADPH |
| GAPDH-q-R | 5'-AACCTTCTTGGCACCACCCT-3' | Relative qRT-PCR analysis of NbGADPH |
| NtCaM-q-F | 5'-ATGGAAAAGTGTCACCGGCTGAGC-3' | Relative qRT-PCR analysis of NtCaM |
| NtCaM-q-R | 5'-TTCCAAATGCTCCTATCAATTCACTC-3' | Relative qRT-PCR analysis of NtCaM |
| SlCaM-q-F | 5'-GGGTATTTACGTACTTTGATGAGAATGG-3' | Relative qRT-PCR analysis of SlCaM |
| SlCaM-q-R | 5'-TATTCCTCTCTTCCTCCATTCCTTCC-3' | Relative qRT-PCR analysis of SlCaM |
| NbAGO1-q-F | 5'-CTGGCGTGGCTTCTATCAAAGTATT-3' | Relative qRT-PCR analysis of NbAGO1-1 |
| NbAGO1-q-R | 5'-CACCTTTACACCTCTCAGTGCCTTC-3' | Relative qRT-PCR analysis of NbAGO1-1 |
| NbAGO4-q-F | 5'-GGAACTATGACTTCTACCTGTGTGCC-3' | Relative qRT-PCR analysis of NbAGO4-1 |
| NbAGO4-q-R | 5'-AACTTCATCCATTGTCCAACTTGTGT-3' | Relative qRT-PCR analysis of NbAGO4-1 |
| NbDCL1-q-F | 5'-TCTTTACATACACAGATCTCCCC-3' | Relative qRT-PCR analysis of NbDCL1 |
| NbDCL1-q-R | 5'-ACCTGGTTTTGATAGTTCATTTT-3' | Relative qRT-PCR analysis of NbDCL1 |
| NbDCL2-q-F | 5'-GAAGGCAGAAGACTAAAAGTAAGAGG-3' | Relative qRT-PCR analysis of NbDCL2 |
| NbDCL2-q-R | 5'- CTACAAGCAGAGAAGGATCATGGAAC-3' | Relative qRT-PCR analysis of NbDCL2 |
| NbDCL3-q-F | 5'-GAGTCAACAGAGCGTAAATCCAAGTC-3' | Relative qRT-PCR analysis of NbDCL3 |
| NbDCL3-q-R | 5'-CTGTCGTTCTAGCTCATACAGCATGA-3' | Relative qRT-PCR analysis of NbDCL3 |
| NbDCL4-q-F | 5'- GCAAAGACTGAAGTTAGGTGTAGCAA-3' | Relative qRT-PCR analysis of NbDCL4 |
| NbDCL4-q-R | 5'-TTTCATCAGCAAGTGGAATAAAGATT-3' | Relative qRT-PCR analysis of NbDCL4 |
| NbSGS3-q-F | 5'-GTTCCTCCTGCTCTGAAGAATGG-3' | Relative qRT-PCR analysis of NbSGS3 |
| NbSGS3-q-R | 5'-GGGCAGTGCCACTGTCGTTCAGG-3' | Relative qRT-PCR analysis of NbSGS3 |
| NbRDR1-q-F | 5'-GACAACTTCCAAAATCTCCATCCA-3' | Relative qRT-PCR analysis of NbRDR1 |
| NbRDR1-q-R | 5'-TTCTCCCACTCCTCATCAACAAAA-3' | Relative qRT-PCR analysis of NbRDR1 |
| NbRDR2-q-F | 5'-GGCACCAACTCATTCTAACCTC-3' | Relative qRT-PCR analysis of NbRDR2 |
| NbRDR2-q-R | 5'-CCAACCTATAACACTCCCCCTT-3' | Relative qRT-PCR analysis of NbRDR2 |
| NbRDR6-q-F | 5'-GTATGCGGAACTTGAATAGGAATGTG-3' | Relative qRT-PCR analysis of NbRDR6 |
| NbRDR6-q-R | 5'-GGTCTTCTGCAAAAAACCAGGCAGA-3' | Relative qRT-PCR analysis of NbRDR6 |
| NbSu-q-F | 5'-GCTTCTACACCCTTGTCTTCTCG-3' | Relative qRT-PCR analysis of Nb-Su |
| NbSu-q-R | 5'-CCCCTATCACCCATTATCATCAC-3' | Relative qRT-PCR analysis of Nb-Su |
